# Task-based functional neuroimaging in infants: a systematic review

**DOI:** 10.3389/fnins.2023.1233990

**Published:** 2023-08-16

**Authors:** Kofi Agyeman, Tristan McCarty, Harpreet Multani, Kamryn Mattingly, Katherine Koziar, Jason Chu, Charles Liu, Elena Kokkoni, Vassilios Christopoulos

**Affiliations:** ^1^Department of Bioengineering, University of California, Riverside, Riverside, CA, United States; ^2^Neuroscience Graduate Program, University of California, Riverside, Riverside, CA, United States; ^3^Orbach Science Library, University of California, Riverside, Riverside, CA, United States; ^4^Division of Neurosurgery, Children’s Hospital Los Angeles, Los Angeles, CA, United States; ^5^USC Neurorestoration Center, University of Southern California, Los Angeles, CA, United States; ^6^Department of Neurological Surgery, University of Southern California, Los Angeles, CA, United States

**Keywords:** task-based neuroimaging, functional neuroimaging, infants (0 to 24 months), brain development, motor development, motor system

## Abstract

**Background:**

Infancy is characterized by rapid neurological transformations leading to consolidation of lifelong function capabilities. Studying the infant brain is crucial for understanding how these mechanisms develop during this sensitive period. We review the neuroimaging modalities used with infants in stimulus-induced activity paradigms specifically, for the unique opportunity the latter provide for assessment of brain function.

**Methods:**

Conducted a systematic review of literature published between 1977–2021, via a comprehensive search of four major databases. Standardized appraisal tools and inclusion/exclusion criteria were set according to the PRISMA guidelines.

**Results:**

Two-hundred and thirteen papers met the criteria of the review process. The results show clear evidence of overall cumulative growth in the number of infant functional neuroimaging studies, with electroencephalography (EEG) and functional near-infrared spectroscopy (fNIRS) to be the most utilized and fastest growing modalities with behaving infants. However, there is a high level of exclusion rates associated with technical limitations, leading to limited motor control studies (about 6% ) in this population.

**Conclusion:**

Although the use of functional neuroimaging modalities with infants increases, there are impediments to effective adoption of existing technologies with this population. Developing new imaging modalities and experimental designs to monitor brain activity in awake and behaving infants is vital.

## Highlights

- Practical and technical limitations, mainly associated with non-compliance, motion artifacts, and data acquisition are exacerbated in infant imaging.
- These constraints lead to high rates of participant exclusions and have resulted in a limited number of studies investigating motor systems in early infancy.
- Overcoming these challenges will require improvements to existing technology, or the development of a novel imaging technique that is age appropriate, portable, cost effective, and less susceptible to motion artifacts, while maintaining high spatiotemporal resolution.

## 1. Introduction

Early human development is marked by robust and rapid changes in brain structure and function. During the first two years of life, essential neuronal transformations commence and complete to establish lifelong abilities (Gilmore et al., 2018). In the first year of life, in particular, there is a 106% increase in the cortical and subcortical grey matter volume (Gilmore et al., 2012). Axonal maturation and myelination of the white matter occur during this period before continuing at a slower pace thereafter (Gao et al., 2009). Furthermore, synaptogenesis – the formation of connections between neurons that plays a vital role in learning, memory formation, and adaptation – continues from the prenatal period and peaks during the first years of life (Tierney and Nelson, 2009). In the visual and prefrontal cortexes, maximum synapse density per neuron formation and rapid acceleration in synaptogenesis is attained between two and four months of age (Huttenlocher, 1990). Overall, the infant’s brain structural appearance by the age of two years is suggested to be comparable to that of an adult’s (Matsuzawa, 2001; Paus et al., 2001).

The ability to monitor the development of the brain during this period is crucial for diagnostic and therapeutic purposes. Structural neuroimaging modalities allow for monitoring of whole-brain volumes with high spatial resolution (Dickerson, 2007). For example, magnetic resonance imaging (MRI) allows for observation of brain morphometry, by providing information on volumetric changes, structural covariance networks, and developmental neurogenesis in the cerebral cortex, hippocampus, and cerebellum (Peterson et al., 2003; Mosconi et al., 2006; Dubois et al., 2014). Nonetheless, understanding the neural mechanisms underlying cognitive functions requires the use of functional neuroimaging modalities (Yamada et al., 1997; Heep et al., 2009; Raschle et al., 2012; Bednarz and Kana, 2018; Cusack et al., 2018). Resting-state and task-based represent the two most common experimental paradigms in functional neuroimaging.

Resting-state measures spontaneous brain activity to study how distributed networks of brain regions, which are functionally connected, are co-activated in the absence of an explicit task (Barkhof et al., 2014). Resting-state neuroimaging has permitted the discovery of distinct patterns of brain connections known as resting state networks (RSNs) and is considered one of the most prevalent neuroimaging techniques since it does not require the subject to perform specific behavioral tasks (Smith et al., 2013). The popularity of resting-state studies in pediatric populations over the past years has furthered our understanding of the development of functional brain networks, especially the large-scale organization of the developing brain (Uddin, 2010). Additionally, resting-state neuroimaging has provided significant insight into differences in brain network characteristics, such as functional connectivity, network topology, and network asymmetry, between typically developing children and atypical young populations, such as early preterm infants and children with neurological disorders (Hu et al., 2020).

Task-based neuroimaging, on the other hand, measures brain activity changes between resting and task-stimulated states to identify brain regions and/or distributed networks that are functionally involved with the specific action (Di et al., 2013; Sumner et al., 2018). A unique property of functional neuroimaging is its use with awake and behaving participants, which provides opportunities for detecting goal-directed task effects and associated regional neuronal activations. A range of modalities are currently available for this purpose. For instance, task-based functional magnetic resonance imaging (fMRI) has been extensively used over the past years in adult populations to understand how higher cognitive functions, such as working memory (McNab and Klingberg, 2008), language processing (Binder et al., 1997), visual attention (Mukai et al., 2007), and loss aversion (Tom et al., 2007), can be localized to specific brain regions. Recently, functional near-infrared spectroscopy (fNIRS) was introduced as a tool for imaging cortical activity with a greater tolerance for head motions compared to that of fMRI (Nishiyori, 2016). Similar to fMRI, fNIRS measures the hemodynamic response to neural activity. Unlike fMRI, fNIRS does not provide whole brain measurements and is only capable of detecting hemodynamic changes that occur within the first few centimeters of cortical tissue (Scarapicchia et al., 2017). Other neuroimaging modalities, such as electroencephalography (EEG) (Darvas et al., 2004; Sohrabpour et al., 2020) and magnetoencephalography (MEG) (Baillet, 2017; Fred et al., 2022) measure non-invasively the electrical potential and the magnetic fields of the brain areas, respectively, and have been also used in pediatric neuroimaging for both clinical and research purposes (Gondo et al., 2001; Bell and Wolfe, 2007). Both provide whole brain measurements with better temporal resolution than fMRI but suffer from low spatial resolution and poor localization of signal sources; therefore, presenting a challenge for decoding specific areas of neuronal activity. Despite significant advancements in the application of the aforementioned functional neuroimaging techniques in populations under the age of two years, certain challenges that require further attention and resolution yet remain (Bookheimer, 2000; Born et al., 2002; Coles and Li, 2011; Gilmore et al., 2018).

A common theme in the preponderance of infant functional neuroimaging studies is that they are conducted with asleep or sedated participants (Bookheimer, 2000; Born et al., 2002; Coles and Li, 2011; Gilmore et al., 2018). While this experimental design has provided significant insights about resting-state brain activity patterns and processes, it limits the stimulation and behavioral paradigms that can be used to study neural correlates of developmental systems. Several review papers have explored the use of neuroimaging modalities in pediatric populations (Parikh, 2016; Gilmore et al., 2018; Mohammadi-Nejad et al., 2018; Azhari et al., 2020) with various objectives in each study – i.e., from describing the use, advantages, and disadvantages of selected modalities (Parikh, 2016; Azhari et al., 2020), to resting-state neuroimaging (Mohammadi-Nejad et al., 2018) and general structural and functional development of the infant brain (Gilmore et al., 2018). To our knowledge, there are no systematic reviews that have specifically focused on the use of functional brain imaging with awake and behaving infants. Our systematic review explores the use of functional neuroimaging modalities in the first two years of life, with stimulus-induced activity paradigms being the core of this examination. It is important to note that this systematic review does not aim to go into detail on how each of the functional neuroimaging modalities works, what hardware components they consist of, how the brain signal is recorded and processed, and so on. There are already excellent reviews that explain all details of classical neuroimaging modalities (Wilke et al., 2003; Papadelis and Chen, 2020; Xie and Nelson, 2021; Gallagher et al., 2023). Instead, the objective of our work is to reveal: (1) what modalities have been utilized in infant neuroimaging to investigate different brain mechanisms specifically involved in task-based experiments, and (2) the challenges and limitations associated with the use of these modalities to acquire brain signals from the infant brain. Lastly, we introduce functional ultrasound imaging (fUSI), a novel modality with excellent spatiotemporal resolution, penetration depth, and compatibility with freely-moving participants, as a complementary modality for use in infant task-based functional neuroimaging.

## 2. Methods

### 2.1. Search strategy

A comprehensive computerized search of five major databases (PubMed, Medline OVID, IEEE Xplore, Web of Science, ProQuest) was conducted for all years up to and including October 2021, according to the PRISMA guidelines (Page et al., 2021). The search employed a combination of relevant MeSH terms, keywords and word variants (see Appendix), such as the following: (“Functional_Neuroimaging”[MeSHTerms])_AND_(“Analytical _Diagnostic_and_Therapeutic_Techniques_and_Equipment_Category”[MeSHMajorTopic])_AND_(“Infant”[MeSHTerms])_AND_(“Cerebrum”[MeSHMajorTopic]_OR_“Cerebellum” [MeSHMajorTopic]). Search results were exported from each database into a dedicated EndNote library.

### 2.2. Exclusion criteria

Journal articles were excluded if: (i) they were not peer-reviewed and/or published in English; (ii) they involved study participants outside of the targeted age range (0–2 years of age); (iii) they described resting-state only experiments- no stimulus (passive or active) was presented to the participants; and (iv) they reported findings on the use of structural brain imaging only. The articles in which mixed-aged participants were reported, only information specific to infants were selected for data extraction. Retrospective and case study reports were included unless they violated the exclusion criteria. Lastly, systematic reviews were excluded.

### 2.3. Study selection

The abstracts of articles resulting from the database search were screened independently by at least two reviewers. Full-text articles resulting from the abstract screening were reviewed independently by four reviewers, ultimately leading to the final set of articles from which data were extracted. Inconsistencies and disagreements were discussed by all reviewers to reach consensus.

### 2.4. Data extraction

The final set of articles were reviewed to extract information on: (i) publication (author, year); (ii) study participants (age, gender and developmental status of participants, reasons for excluding participants from study, awareness state of participants during brain imaging acquisition); (iii) study design (type of study, number of brain imaging acquisition sessions in the study, purpose for brain imaging acquisition in the study); and (iv) experimental design (type of stimulus applied to the participants, timing and order in which stimuli were presented to the participants, functional system of the cerebral cortex that was targeted via stimulation of participants, brain imaging modalities used in the study).

## 3. Results

### 3.1. Selected studies

**Figure 1:**
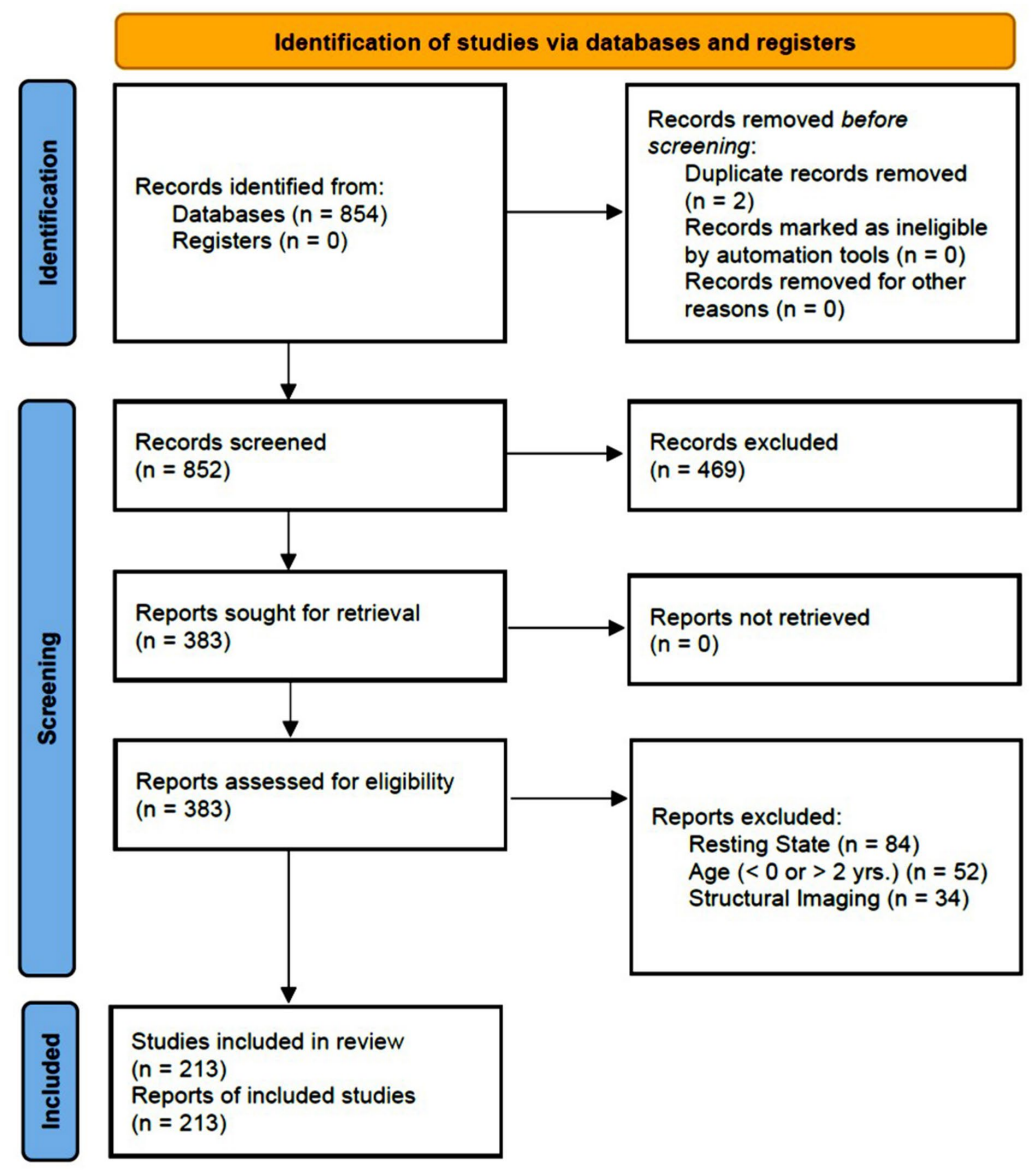
Flow chart illustrating the different phases of the systematic review.

The database search produced a total of 854 articles. Initial screening of abstracts resulted in the exclusion of 469 articles. At this stage, articles were excluded if at least one of the exclusion criteria was mentioned in the abstract; otherwise, the articles passed to the next stage of the review process. At the next stage, the full-text of the remaining 385 articles were reviewed and 170 more articles were excluded. Ultimately, 213 articles were included in this systematic review (See the “Search Strategy and Results” in Appendix). Figure 1 illustrates the review process along with the exclusion criteria.

### 3.2. Characteristics of participants and experimental studies

**Figure 2:**
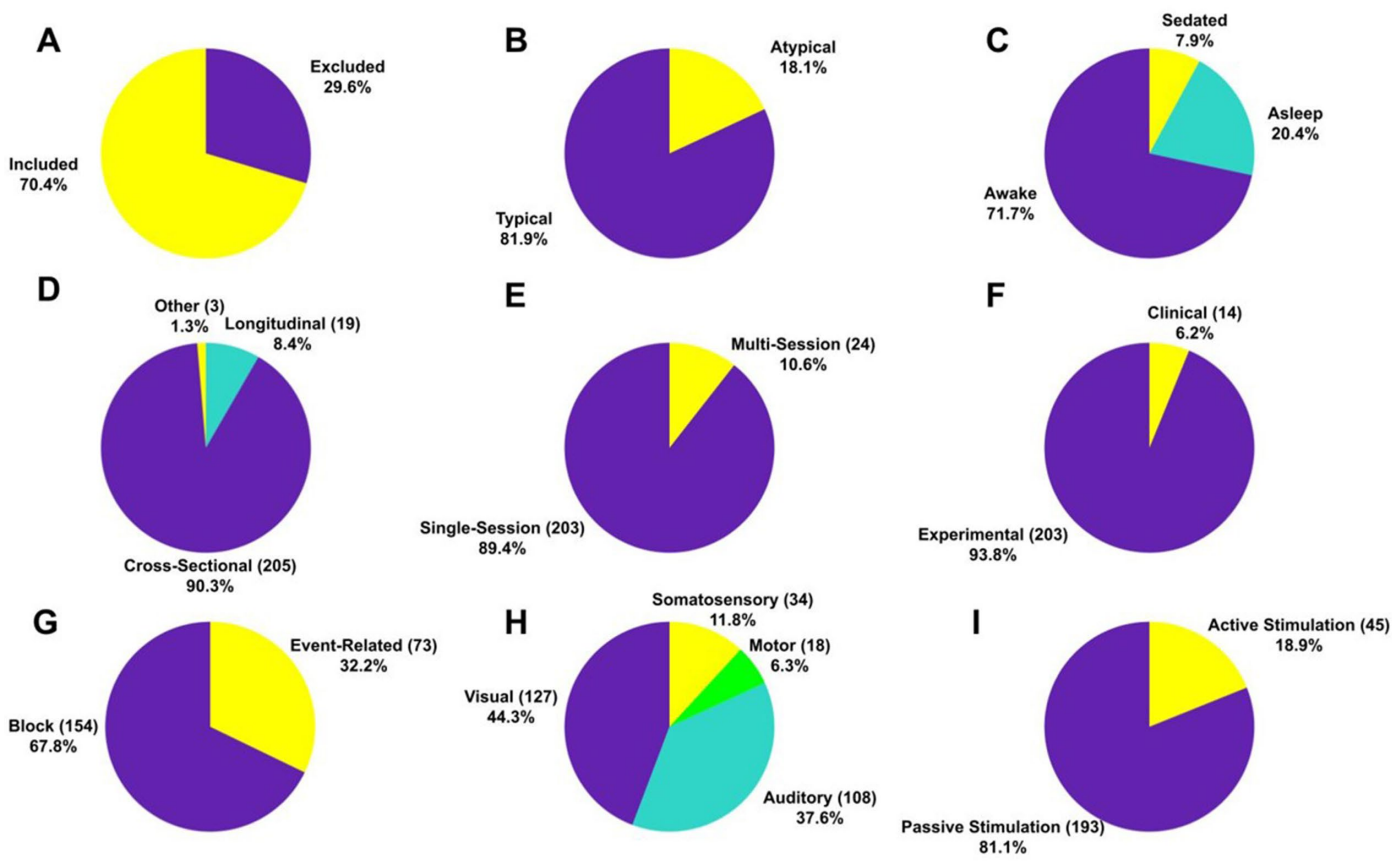
Characteristics of participants and experimental studies. Proportion (and absolute number) of **(A)** participants that included and excluded in the studies, **(B)** typical and atypical participants in the studies, **(C)** participants being sedated, asleep and awake during image acquisition, **(D)** cross-sectional and longitudinal studies, **(E)** single and multi-sessions studies, **(F)** clinical and experimental studies, **(G)** blocked and event-related design studies, **(H)** studies that explored motor, visual, somatosensory and auditory systems of the infant brain, **(I)** studies with active and passive stimulation.

A total of 7461 infants (Mean = 35.53 per study, SD = 35.59) under two years of age (291 preterm, 7,170 full-term) participated in the studies. Out of the 75.6% of articles that reported sex, female and male infants accounted for 47.4 and 52.6%, respectively. The exclusion rate was 29.6% (Mean = 19.34% and SD = 28.53%) across all studies (Figure 2A). Most of the participants (81.9%) were healthy (i.e., of typical development), whereas the remaining had atypical patterns of development due to underlying neurological conditions (Figure 2B). Regarding the conscious state, 71.7% of the participants were awake, 20.4% were asleep and the rest were anesthetized (Figure 2C).

The majority of brain imaging studies were cross-sectional (90.3%), involved a single session (89.4%), conducted for non-clinical (i.e., experimental) purposes (93.8%) (Figures 2D–F), and most of these studies were conducted in block design (67.8%) (Figure 2G). More than three-fourths of the studies explored the visual system ( 44.3% ) and the auditory system ( 37.6% ), whereas about 12% investigated the somatosensory system (Figure 2H). Surprisingly, only 6.3% of the studies focused on the motor system indicating that motor functions in the infant brain have been understudied. Finally, passive stimulation modes, in which infants did not have to perform any voluntary action, were mostly utilized (81.1%) (Figure 2I).

### 3.3. Functional brain imaging modalities in infants

**Figure 3:**
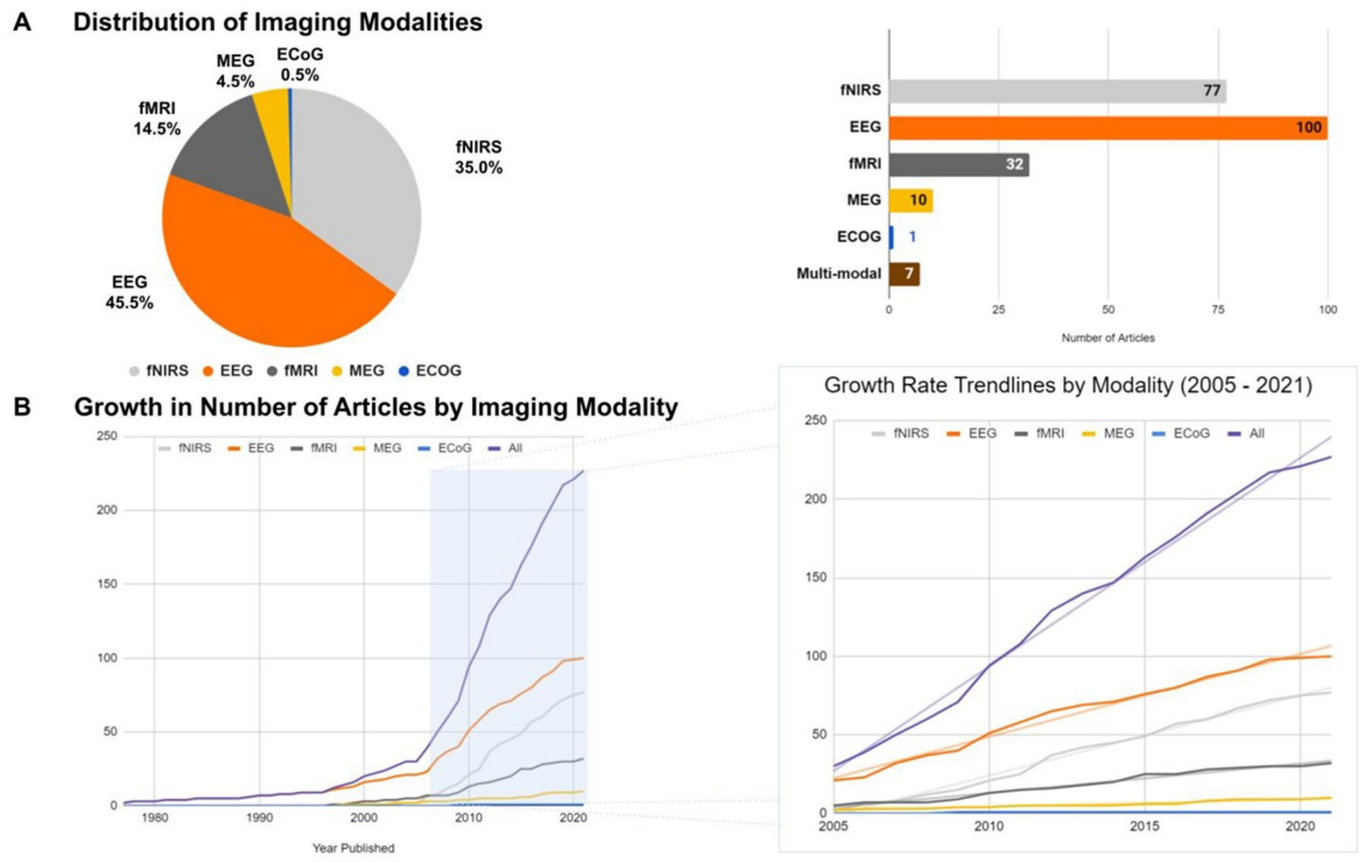
**(A)** Relative proportion (left) and number (right) of articles reporting the use of various brain imaging modalities with infants (fNIRS, EEG, fMRI, MEG, and EcoG). **(B)** Growth rates in the number of articles reporting each imaging modality across time (left) with an emphasis in the last 16 years (right).

Brain imaging research in infants has been rapidly increasing over the past years. This review revealed that the most highly reported modalities are EEG (45.45%) and fNIRS (35.0%) followed by fMRI (14.55%), MEG (4.54%) and ECoG (0.5%) (Figure 3A). Near-Infrared Optical Tomography (NIOT) and Diffuse Optical Tomography (DOT), two optical neuroimaging modalities that, like fNIRS, use near-infrared light to measure tissue oxygenation, resulting in a hemodynamic measure of neuronal activity, were reported in four articles (Taga and Asakawa, 2007; Nishida et al., 2008; Liao et al., 2012; Shekhar et al., 2019). These techniques were grouped with the articles on fNIRS for simplification. Additionally, we found that 3% of the current brain imaging studies in infants utilized a combination of up to two imaging modalities. The complementary nature of EEG and fNIRS was combined in half of these studies to get a high temporal and spatial resolution at the same time; and EEG was generally the most frequently-used modality in combination with others (fMRI, fNIRS, and MEG), followed by fMRI (paired with EEG, ECoG, and MEG).

The total number of studies published in this field before the year 2005 account for about 14% (i.e., 30 articles) of the total published articles, wherein EEG can be considered as the main functional imaging technique for monitoring the infant brain. A breakthrough in functional brain imaging for infants occurred around 2005, where the average publication rate increased from 2.1 articles per year (between 1995 to 2005) to 11.6 articles per year. In particular, the annual cumulative average growth rates from 2005 to 2021 was 5.11 ( $R^{2}$ = 0.99) – fNIRS, 5.26 ( $R^{2}$ = 0.99) – EEG, 1.87 ( $R^{2}$ = 0.98) – f MRI and 0.48 ( $R^{2}$ = 0.94) – MEG [*p* < 0.01] (Figure 3B). These results revealed that EEG is the preponderant technique for studying the infant brain, but fNIRS has seen a surge in popularity within this field from 2005 to present day.

### 3.4. Exclusion rates in brain imaging modalities for infants

**Figure 4:**
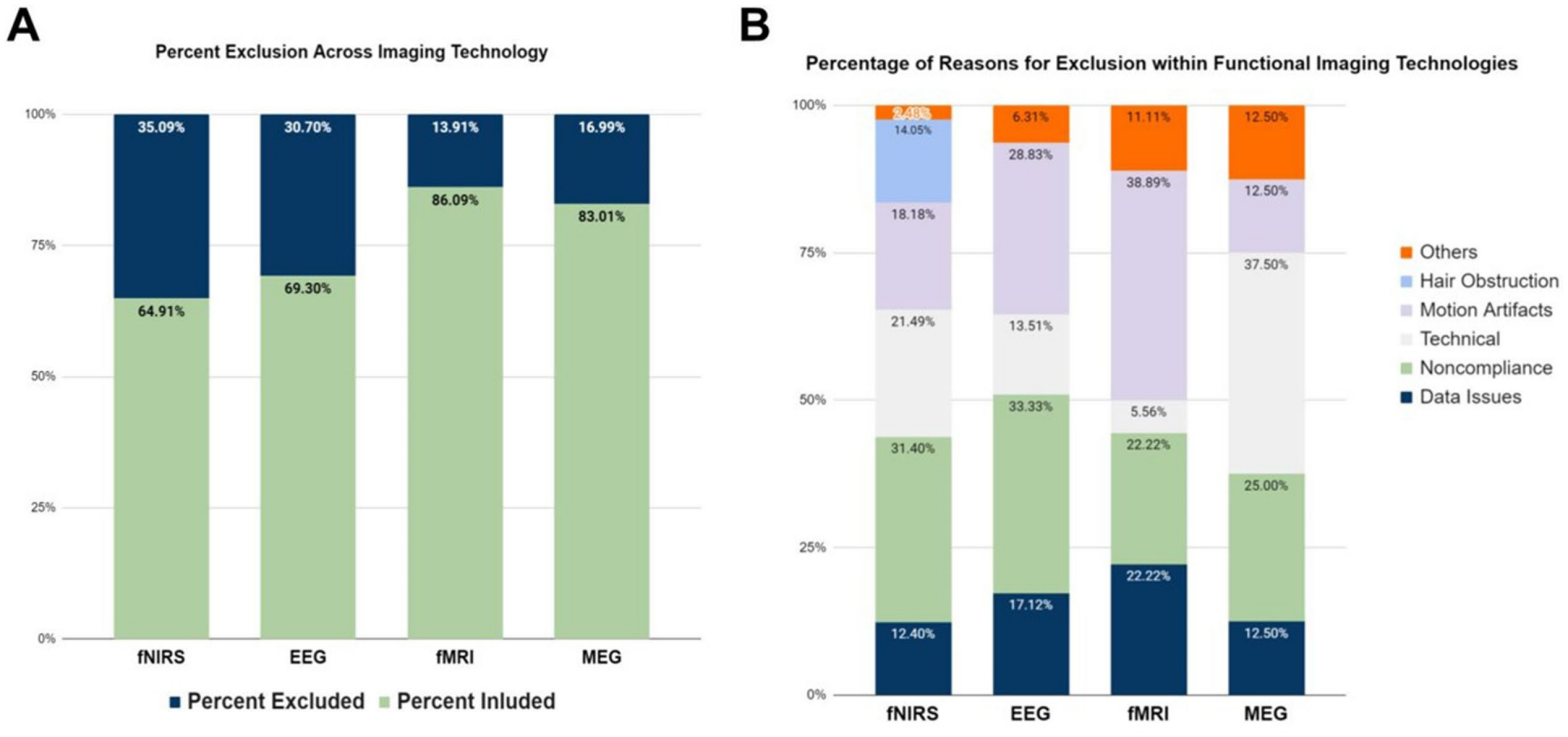
Exclusion reasons for different brain imaging modalities. **(A)** Percentage of participants excluded in the studies per brain imaging modality. **(B)** Reported reasons for participant exclusion across per brain imaging modality. Data on ECoG are not displayed, since there was only one study in the literature.

Among the brain imaging modalities, fNIRS and EEG reported the highest exclusion rates (35.09 and 30.70% respectively), whereas fMRI studies had the lowest exclusion rate (13.91%). Although these findings may appear counter-intuitive, since fMRI is traditionally considered the most susceptible imaging technique to motion artifacts, it can be attributed to the use of fMRI with a relatively higher number of sedated and asleep infants (results section E). Lastly, MEG studies had the second lowest exclusion rate observed (16.99%). These relationships can be seen in Figure 4A.

The most highly-reported reasons for participant and/or data exclusion were technical issues, motion artifacts, noncompliance of participants (e.g., fussiness, inattentiveness), and data issues (Figure 4B). fNIRS seems to be more sensitive to all issues reported, and was the only imaging modality that excluded participants due to hair obstruction. It is often the case that signal-to-noise ratio (SNR) in fNIRS is low due to hair obstruction that causes poor contact between the optical fibers that deliver and collect near infrared (NIR) light to and from the infant scalp (Orihuela-Espina et al., 2010). The highest percentage of excluding participants due to motion artifacts were reported in fMRI studies (38.89%), as it was expected since head and body movement artifacts are an inherent problem to magnetic resonance imaging (MRI) technology. fNIRS (18.18%) and EEG (28.83%) studies also frequently reported data exclusions due to motion artifacts. One interesting finding is that fMRI studies reported the lowest rate of noncompliance issues, which could be explained by the fact that most of them involve sedated or asleep infants.

### 3.5. Experimental and methodological aspects of brain imaging modalities in infant populations

**Figure 5:**
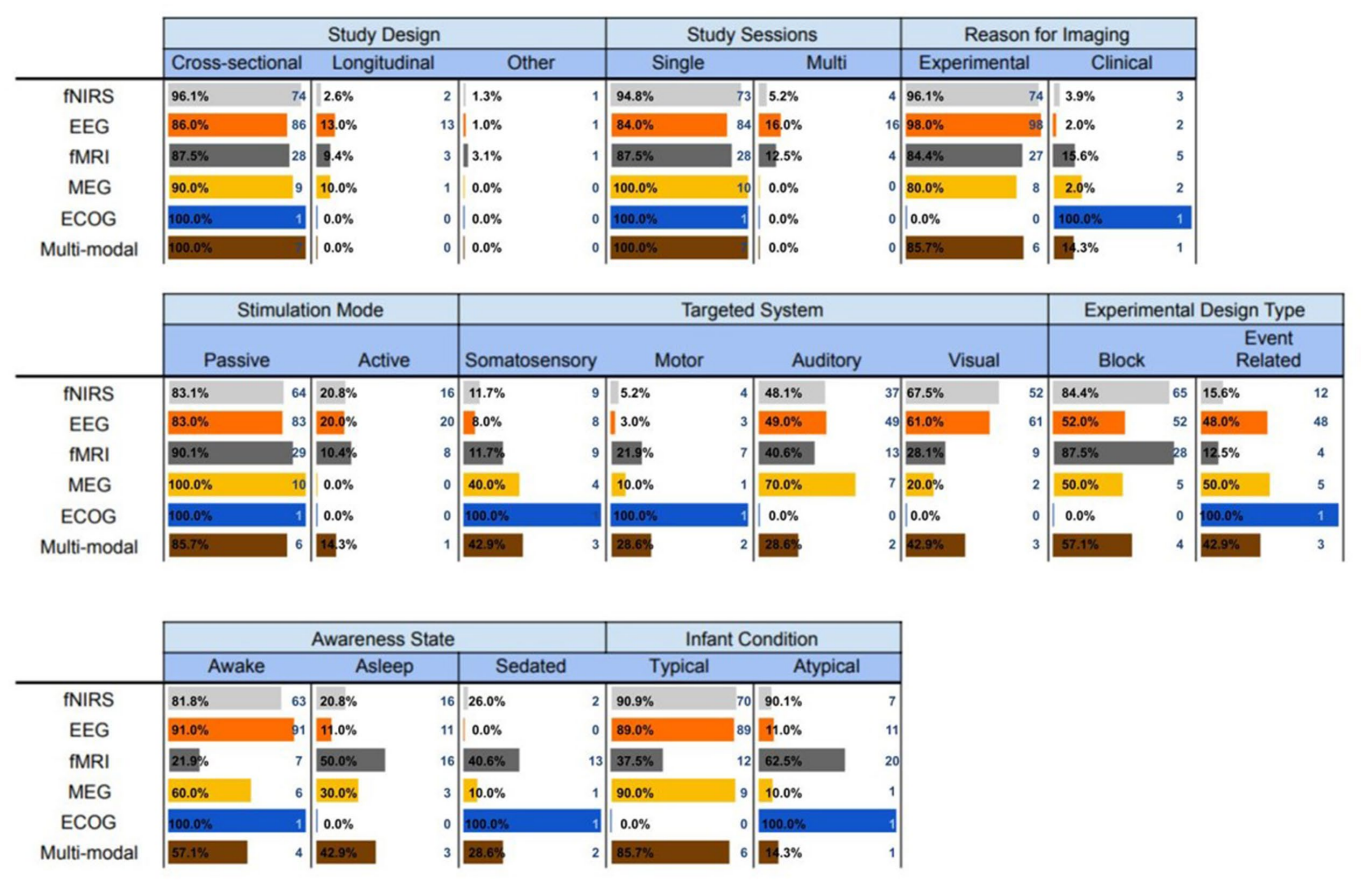
Experimental and methodological characteristics of the brain imaging modalities utilized with infants.

In this section, we describe in detail the experimental and methodological aspects of each of the brain imaging modalities that were utilized in the infant studies. The findings are summarized in Figure 5.

#### 3.5.1. Functional near-infrared spectroscopy

Although fNIRS is a relatively new optical imaging technique, our analysis showed that it is rapidly becoming one of the leading neuroimaging modalities for infant populations. Almost all the fNIRS studies acquired brain activity using cross-sectional design within single sessions, and only 4 out of 77 studies performed in multiple sessions. Additionally, almost all studies were experimental (74 out of 77 studies), with only 3 clinical trials. About 80% of the studies utilized a passive stimulation mode, in which participants did not have to respond to external stimuli. Instead, they passively observed (visual studies), listened (auditory studies) and felt (somatosensory studies) stimuli, or performed passive movements driven by external devices (motor studies). fNIRS was primarily used in studies that addressed questions related to the auditory ( 48.1% ) and visual ( 67.5% ) brain systems, whereas the rest of them explored the somatosensory ( 11.7% ) and motor ( 5.2%) brain systems. Additionally, fNIRS studies were mainly performed in block design experiments ( 84.4% ), a non-surprising finding given that blocked design dominated the early years of adult functional neuroimaging (Huettel et al., 2009). Finally, the participants were awake in about 82% of the studies, and almost all of them include typical infants, except for 7 studies that measured brain activity in atypical populations (Isobe et al., 2001; Kusaka et al., 2004; Nishida et al., 2008; KUSAKA et al., 2011; Bembich et al., 2016; Braukmann et al., 2018; Lloyd-Fox et al., 2018).

#### 3.5.2. Electroencephalography

Electroencephalography (EEG) was one of the first imaging modalities introduced in infants, and remains today the most popular overall technique for this purpose. Similar to fNIRS, the vast majority of EEG studies were also cross-sectional ( 86% ), although EEG has been presented in most of the longitudinal brain imaging studies in infant populations – i.e., almost 70 % of the longitudinal brain imaging studies (13 out of 19 total studies) in infants were conducted using EEG. It has been also largely used in single sessions ( 84.0% ), although two-thirds of studies which were performed in multiple sessions (i.e., 16 out of 24) utilized EEG. Interestingly, EEG has only been used in 2 clinical trials, accounting for only 1% of the total EEG studies to date. EEG studies were designed using mainly passive stimulation modes ( 83.0% ), to predominantly explore the visual ( 61.0% ) and the auditory system ( 49.0% ). Surprisingly, EEG has only been used in 3 motor control studies. Furthermore, about half of the studies ( 52% ) used block design and the rest ( 48% ) event-related design to perform the tasks. It is also worth mentioning that EEG was the only imaging modality that did not involve sedated infants. More than 90% of the studies were conducted in awake infants and the rest in asleep infants. Similar to fNIRS, almost all EEG studies were conducted in typical populations, with only a few exceptions (Haartsen et al., 2019).

#### 3.5.3. Functional magnetic resonance imaging

The third most popular neuroimaging technology also shares many common experimental and methodological characteristics with the two aforementioned modalities. About 85% of the fMRI studies were primarily cross-sectional, and performed in single sessions for experimental purposes. The vast majority of the studies used passive stimulation modes ( 90.6% ) and conducted on block design ( 87.5% ) consistent with the EEG and fNIRS infant neuroimaging studies. However, unlike EEG and fNIRS, fMRI has been used for studying the brain mechanisms associated with the somatosensory ( 28.13% of the studies), motor systems ( 21.88% ), auditory ( 40.63% ) and the visual ( 28.13% ) systems. fMRI is especially dominant in the field of motor control in infant populations, in which 7 out of 16 motor studies that used a single imaging modality utilized fMRI, a surprising finding given its susceptibility to motion artifacts. This is most likely due to the fact that the majority of the fMRI experiments are performed on sedated or asleep infants – only 7 out of 32 fMRI studies involved awake participants. This was another major difference between fMRI and fNIRS and EEG studies, which were mostly conducted in awake infants. A noteworthy finding was that fMRI was used more often on atypical populations as opposed to typical (12 and 20 studies, respectively), indicating that fMRI is the major neuroimaging modality for atypical populations (i.e., more than 62 % of studies).

#### 3.5.4. Magnetoencephalography

The least popular non-invasive neuroimaging technology for infant populations is MEG. All MEG studies were cross-sectional (with just one exception), conducted in single sessions, for experimental purposes (two exceptions), using passive stimulation modes. The majority of these studies investigated the auditory (70 % ), somatosensory (40 % ), and visual (20%) systems, with only one exception that investigated the motor system (Narayana et al., 2015). Using MEG technology to explore the visual system of infants is a relatively recent phenomenon, with only two studies examining this topic, both within the past 4 years. Half of the studies were conducted in block design – i.e., 5 event-related and 5 block design MEG studies. One of interesting findings was that the infants were awake in most studies (60%), whereas in the rest of them were either sedated or asleep. Finally, except for one study (Stephen et al., 2017), the participants were all typical infants with no neurological or other deficits.

#### 3.5.5. Electrocorticography

The systematic review analysis identified one study that used ECoG to measure brain activity in sedated and atypical infants. ECoG is an invasive technique that is frequently used clinically to map epileptogenic regions of the brain (Reif et al., 2016). It records electrical activity in the brain by placing a grid of electrodes in direct contact with the cerebral cortex. This one study was cross-sectional that was conducted in a single session for clinical purposes. A passive range of motions were implemented in an event-related design to identify the motor cortex in sedated infants.

#### 3.5.6. Multi-mode imaging modalities

Although most neuroimaging studies in infant populations utilized one of the aforementioned techniques, we found 7 studies that measured brain activity by combining data obtained from two neuroimaging modalities. EEG was the most frequently-used modality that was combined with one of the alternative technologies; being used most often in conjunction with fNIRS (Grossmann et al., 2008; Watanabe et al., 2010; Biallas and Trajkovic, 2012; Verriotis et al., 2016). All multimodal neuroimaging (MN) studies were cross-sectional that performed in single sessions for mostly experimental purposes ( 85.7% ). They also predominantly used passive stimulation modes ( 85.7% ), with just one exception (Grossmann et al., 2008), and they have been utilized in studying all brain systems. Four of the studies were implemented in block design and three in event-related design using awake ( 57.14% ), asleep ( 42.86% ) and sedated ( 28.57% ) mainly in typical infants, with only one study in which the infant population was atypical (Ogg et al., 2009).

## 4. Discussion

The first two years of life are characterized by rapid neurological transformations leading to consolidation of crucial lifelong cognitive, perceptual, memory, and behavioral abilities (Slater et al., 1988; Huttenlocher, 1990; Matsuzawa, 2001; Paus et al., 2001; Taga et al., 2002; Johnston et al., 2009; Gilmore et al., 2012, 2018). Advances in neuroimaging modalities have started to uncover structural, resting state functional brain networks and activity patterns in both animals and humans. However, the neural mechanisms sub-serving goal-directed motor action and control in awake infants remain unclear and understudied. To our knowledge, this is the first systematic review of literature on the use of functional imaging modalities to study the infant brain in research and clinical settings. The findings from this search revealed the need for effective functional assessment of the brain of awake and behaving infants.

### 4.1. The motor system is understudied in infancy

A main finding from this systematic review is that the neural mechanisms of motor control in infancy have not been adequately examined. The lack of motor-related tasks during brain imaging acquisition is evident across all functional brain imaging modalities. While a general migration toward fNIRS and EEG use is observed over the years, the application of the latter to study the motor system in awake and behaving infants remains limited. Only four studies employed fNIRS and three studies employed EEG to examine the motor system in task-based experiments; rather the majority of the studies focused on the auditory, visual, and somatosensory systems. This finding may be associated to various factors, as this review reveals. Motion artifacts seems to be one of the most common reasons across all the main imaging modalities (except MEG). Even fNIRS and EEG, which were designed to assess brain activity, are not immune to inherent motion artifacts and technical challenges. This factor may have contributed to the predominant examination of the non-motor systems since they do not require considerable motion or movement of participants.

Nevertheless, assessing the development of the motor system in awake and behaving infants is critical. The study of early motor patterns and system responses to environmental stimuli can inform the prediction of neuromotor outcomes in infants at risk for developmental delays. An example is the examination of the presence, quality, and quantity of general movements, such as the fidgety movements that seem to be the primary movement pattern in awake infants between three and five months of age (Prechtl and Hopkins, 1986). The complexity and variability of these movements have been found to be lower in infants later diagnosed with cerebral palsy compared to their typically developing peers (Einspieler and Prechtl, 2005; Skiöld et al., 2013). Clinical and behavioral standardized assessments, such as the Test of Infant Motor Performance (Campbell et al., 1995) and the Hammersmith Infant Neurological Examination (Hay et al., 2018), provide such information and are used as screening tools to predict neuromotor outcomes in infants at risk for motor delays (Noble and Boyd, 2012). When these assessments are combined with brain imaging techniques, however, a better understanding of neurodevelopment and a prediction of neuromotor outcomes can be obtained (Skiöld et al., 2013; Setänen et al., 2014). Currently, these assessments are predominantly combined with structural brain imaging techniques, such as MRI, which cannot provide information about the functional organization of the motor system. Our results thus indicate a need for functional imaging modalities that allow for the assessment of the motor system in awake and behaving infants.

### 4.2. Passive stimulation modes dominate infant neuroimaging research

One of the main findings in this systematic review is that most brain imaging studies use passive stimulation modes (about 80 % total) to explore the neural mechanisms of cognitive functions in infants. One of the reasons that active stimulation modes are not popular in this population is that infants, due to immature age, easily get fussy, often cannot follow or understand instructions, and cannot provide verbal responses.

Although passive stimulation modes have been extensively utilized in infant brain imaging, serious concerns about the validity of the findings can be raised. The main question is whether passive modes evoke the same activation patterns with active modes, in which participants interact and respond to external stimuli. Previous brain imaging studies in young populations and adults showed that it is possible to achieve similar activation patterns within identical brain regions using passive and active stimulation models in sensory (Puce et al., 1995; Yetkin et al., 1995) and motor (Weiller et al., 1996; Ogg et al., 2009; Blatow et al., 2011; Fu et al., 2015) task paradigms. However, these findings contrast strongly with other studies showing that voluntary movements activate different brain areas in active and passive paradigms. For instance, it has been shown that active listening results in stronger inferior frontal activation than more passive paradigms (Vannest et al., 2009; Stoppelman et al., 2013; Yue et al., 2013). Along the same line, it has been reported that passive movements elicit weak and spatially limited brain activation in the subcortico-cortical sensorimotor network (Christensen et al., 2000; Dobkin et al., 2004; Ciccarelli et al., 2005), and in the contralateral primary and secondary somatosensory areas (Mima et al., 1999). On the other hand, active finger movements evoked activation in multiple areas, including the contralateral primary sensorimotor cortex, premotor cortex, supplementary motor area (SMA), basal ganglia and ipsilateral cerebellum (Mima et al., 1999).

One important limitation of passive actions is that they are not accompanied by generation of internal models, which explain how the brain compensates for sensory feedback delays (Beets et al., 2012). Despite the sophistication of our sensory system, raw sensory input is noisy and suffers from long sensory delays. It has thus been proposed that the brain builds internal models to predict the consequences of motor commands before sensory feedback reflects movement execution (Kawato, 1999). Because passive movements are not executed by voluntary motor commands, they lack internal models. Therefore, passive stimulation modes cannot explain how participants plan actions or how they learn from error detection/correction to update internal models (Beets et al., 2012).

Overall, the limited number of studies with an active stimulation component might explain why motor development and control has been understudied in infants. To understand the mechanisms of the motor system in this population, there is a need for the development of new experimental approaches and/or functional imaging modalities that are compatible with active stimulation modes.

### 4.3. Clinical trials are predominantly based on resting-state functional neuroimaging

One of the most important findings in our study was that clinical trials in infant task-based neuroimaging research are extremely limited. In particular, the review analysis revealed only 33 out of the 213 studies involved atypical infants. We need to interpret this result cautiously, because it does not necessarily mean that neuroimaging studies are rarely performed in clinical infant populations. Instead, it could be that most of the clinical functional brain imaging studies in infants are performed in the absence of any external tasks, i.e., resting-state neuroimaging. Resting state neuroimaging is a popular technique in clinical populations, due in part to it having several advantages over task-based neuroimaging (O’Connor and Zeffiro, 2019). The design of the experiment and data acquisition is less complex and therefore more suited for a clinical population, while also simultaneously identifies and evaluates multiple neural systems with a single imaging session. However, the most important advantage is that it allows for a broader sampling of patients, who are not capable of accurately performing tasks, such as young, sedated, paralyzed, comatose or cognitively impaired patients.

Despite the advantages of resting-state neuroimaging, the lack of an external task can impose significant challenges in understanding the pathophysiology of neuropsychiatric diseases. For instance, it is still unclear whether brain networks measured during resting-state exhibit comparable properties to brain networks during task performance. However, recent neuroimaging studies have also reported that cerebral blood flow (CBF) evoked by different tasks account for less than 5 % of the resting-state CBF, suggesting that hemodynamic information of brain networks in resting-state can capture properties of the networks during task performance (Raichle, 2010; Di et al., 2013). Along the same line, studies have shown that task-related activation brain patterns correspond well with the activation brain networks in resting-state (Toro et al., 2008; Smith et al., 2009). On the contrary, it has been argued that brain networks are on “energy saving mode” during the resting-state and exhibit re-configurations during task performance to facilitate global and between systems information transmission (Bullmore and Sporns, 2012). Therefore, even though the functional connectivity of brain networks in tasks conditions and resting-state might be similar, the brain likely has different network configurations to support different task demands (Di et al., 2013). Considering that, it is unclear whether abnormalities in brain networks experience the same behavior in resting-state and task-based neuroimaging. Therefore, to better understand the pathophysiology of motor-related brain diseases, it is critical to study the brain in action through task-based neuroimaging. Finally, although it has been shown that some of the resting-state networks are present already in the infant brain, there are significant differences between adult and infant resting-state networks. For instance, an fMRI study identified 5 similar to the adult brain resting-state networks in the infant brain, but it failed to detect a direct equivalent of the default-mode network (DMN) in the infant brain (Fransson et al., 2007). A possible explanation of the absence of the DMN is the relative immaturity of the infant brain. Therefore, it is reasonable to believe that the DMN, which is primarily involved in self-referential processing, social cognition and self-projection, is not present in the young brain of the infants. These findings provide an extra reason to support that task based experiments are the proper ways to study an infant brain that dynamically evolves over time.

### 4.4. Future directions in infant functional neuroimaging

Our study reveals the need to either improve the current functional neuroimaging technologies and/or to develop new ones that can monitor the brain in awake and behaving infants with high spatiotemporal resolution, sensitivity and penetration depth. It is imperative to use infant-friendly imaging modalities, with light-weight recording devices and faster acquisition algorithms that reduce the time of the experimental session. Also, it is important these imaging modalities to be less sensitive to motion artifacts than the current neuroimaging techniques. Recently, functional ultrasound imaging (fUSI) was introduced as a breakthrough modality that provides a unique combination of excellent spatiotemporal resolution (~100 μm and up to 10 ms), penetration depth (up to 8 cm) and compatibility with freely moving subjects (Macé et al., 2011). Although fUSI typically requires thinned skull surgery or trepanation to enable the penetration of the ultrasound waves, in infants, fUSI images can be acquired non-invasively through the fontanels [median age of the anterior fontanel closure is close to 14 months in term infants, with delays in closure observed in infants developing atypically (Duc and Largo, 1986; Kiesler and Ricer, 2003)]. The first clinical proof-of-concept studies on fUSI in infants were conducted to monitor hemodynamic changes associated with different sleep states and epileptic seizures (Demene et al., 2017; Baranger et al., 2021). In these studies, a light linear array probe (40 gr) with 128 elements and 6 MHz center frequency was used to generate fUSI images with spatial resolution of 250 μm × 250 μm in-plane, a slice thickness of 400 μm and field of view (FOV) 60 mm × 25 mm (depth and width). The penetration depth was sufficient enough to image simultaneously cortical and subcortical regions on the infant brain. Overall, the excellent spatiotemporal resolution, the light probe, the friendly environment, the portability and the high sensitivity to slow blood flow make fUSI technology a promising candidate to monitor brain activity in awake and behaving infants – although this technology has not been evaluated in awake and behaving infants yet.

## 5. Strengths and limitations

There are several strengths and limitations to this review. This is the first systematic review of the current state of imaging modalities to study awake and behaving infants. To progress the field, researchers, engineers, and clinicians should understand the reasons for the current state. This review provides an insight on the needs that are specific to this young population, the reasons that these needs are not currently being met, and lists considerations for future imaging modality development. Second, this review provides information on a broad spectrum of brain systems and modalities with this population. By not restricting the search terms to a specific modality or brain region, this review provides comparative information and highlights the differences among these categories. Nevertheless, the lack of studies on the motor system, which we found in this review, suggests that a follow-up systematic review with search restriction to the motor system could provide significant findings. Lastly, a limitation in our work is the likelihood of omitting possibly relevant papers. However, this is a common limitation among systematic reviews that can occur due to various reasons including, but not limited to, improper assignment of or missing keywords in the article and others.

## 6. Conclusion

In summary, despite the growth of functional neuroimaging in infants, the brain motor mechanisms during development have been understudied. Limitations and trade-offs inherent to current neuroimaging modalities seem to discourage researchers from studying infant brain motor development. This is more evident in atypical populations, which can interfere with early intervention applications for patients are risk for developmental delay. Understanding the mechanisms of motor development, as well as the pathophysiology of neurological developmental disorders requires brain activity monitoring in both typical and atypical behaving infants over extended periods of time. This systematic review demonstrates the need for developing and applying new imaging modalities and experimental designs to monitor brain activity in awake and behaving infants with high spatiotemporal resolution, deep tissue penetration capabilities, and high specificity.

## Data availability statement

The original contributions presented in the study are included in the article/Supplementary material, further inquiries can be directed to the corresponding author.

## Author contributions

EK and VC conceived the study and supervised the research. KK collected the articles. KA, TM, HM, KM, EK, and VC reviewed the articles, extracted, and interpret the data. KA and KM drafted the manuscript with substantial contribution from TM, JC, CL, EK, and VC. All authors edited and approved the final version of the manuscript.

## Funding

Research reported in this publication was supported by the US National Institutes of Health (NIH)-funded Center for Smart Use of Technologies to Assess Real-world Outcomes (C-STAR) under award number P2CHD101899. The funders had no role in study design, data collection and analysis, decision to publish, or preparation of the manuscript.

## Conflict of interest

The authors declare that the research was conducted in the absence of any commercial or financial relationships that could be construed as a potential conflict of interest.

## Publisher’s note

All claims expressed in this article are solely those of the authors and do not necessarily represent those of their affiliated organizations, or those of the publisher, the editors and the reviewers. Any product that may be evaluated in this article, or claim that may be made by its manufacturer, is not guaranteed or endorsed by the publisher.
