# Supplementary material for: Task-based functional neuroimaging in infants: a systematic review
Source: Front Neurosci. 2023 Aug 16;17:1233990. doi: 10.3389/fnins.2023.1233990 (PMC10466897; doi:10.3389/fnins.2023.1233990)
Supplement: Supplementary file 1 [file Data_Sheet_1.PDF]

Citation	IMAGING MODALITY	Number of Participants			Reason for Exclusion	Age (yrs)					Reason for Scan	Participant condition	Subject scan state			Stimulation mode			Targeted System				Scan	Application	
		Recruited	Total	Excluded		< 0	0 - 1	0-2	1 - 2	> 2			Awake	Asleep	Sedated	Passive	Active	Action	Somatosensory	Motor	Auditory	Visual	Frequency	Clinical	Research
Marshall, P. J., & Fox, N. A. (2004). A comparison of the electroencephalogram between institutionalized and community children in Romania. *Journal of Cognitive Neuroscience*, 16(8), 1327–1338. https://doi.org/10.1162/0898929042304723	EEG	216	150	66	various data collection issues, excessive signal data noise, motion	0			used 9month and older breakdown not provided		experiment	healthy	1	0	0	1	0	0	0	0	0	1	single session	0	1
H. Ichikawa, et al. (2019). A longitudinal study of infant view-invariant face processing during the first 3-8 months of life. *NeuroImage*, 186, 817–824. http://ovidsp.ovid.com/ovidweb.cgi?T=JS&PAGE=reference&D=med16&NEWS=N&AN=30529397	fNIRS	14	11	3	incomplete data or excessive motion, hair interference	0	11	0	0	0	experiment only	healthy / typical development	1	0	0	1	0	0	0	0	1	1	6 months (1 session per month)	0	1
Dehaene-Lambertz, G., & Baillet, S. (1998). A phonological representation in the infant brain. *NeuroReport*, 9(8), 1885–1888. https://doi.org/10.1097/00001756-199806010-00040	MEG	35	24	11	noncompliance, data artifacts	0	24	0	0	0	experiment only	healthy / typical development	1	0	0	1	0	0	0	0	1	0	single session	0	1
Bell, M. A. (2012). A psychobiological perspective on working memory performance at 8 months of age. *Child Development*, 83(1), 251–265. http://ovidsp.ovid.com/ovidweb.cgi?T=JS&PAGE=reference&D=med9&NEWS=N&AN=22103396	EEG	50	50	na	na	0	50	0	0	0	experiment only	healthy / typical development	1	0	0	0	1	1	0	0	1	1	single session	0	1
C. J. Wild, et al.(2017). Adult-like processing of naturalistic sounds in auditory cortex by 3- and 9-month old infants. *NeuroImage*, 157, 623–634. http://ovidsp.ovid.com/ovidweb.cgi?T=JS&PAGE=reference&D=med14&NEWS=N&AN=28648887	fMRI	40	13	27	na	6	13	13	0	0	experiment	healthy	0	1	0	1	0	0	0	0	1	0	single session	0	1
J. M. Leppanen M. C. Moulson, V. K. V.-F., & Nelson, C. A. (2007). An ERP study of emotional face processing in the adult and infant brain. *Child Development*, 78(1), 232–245. http://ovidsp.ovid.com/ovidweb.cgi?T=JS&PAGE=reference&D=med6&NEWS=N&AN=17328702	EEG	57	25	32	fussiness, technical issues, movement artifacts	0	15	15	0	10	experiment	healthy	1	0	0	1	0	0	0	0	0	1	single session	0	1
S. Narayana, et al. (2015). Assessing motor function in young children with transcranial magnetic stimulation. *Pediatric Neurology*, 52(1), 94–103. http://ovidsp.ovid.com/ovidweb.cgi?T=JS&PAGE=reference&D=med12&NEWS=N&AN=25439485	fMRI & MEG	na	6	na	na	0	0	4	4	2	experiment	healthy	0	0	1	1	0	0	1	1	0	0	single session	0	1
Huotilainen, M., et al. (2003). Auditory magnetic responses of healthy newborns. *NeuroReport*, 14(14), 1871–1875. https://doi.org/10.1097/00001756-200310060-00023	MEG - mismatch negativity counterpart	12	12	na	na	0	12	0	0	0	experiment only	healthy / typical development	0	1	0	1	0	0	0	0	1	0	single session	0	1
de Haan, M., & Nelson, C. A. (1999). Brain activity differentiates face and object processing in 6-month-old infants. *Developmental Psychology*, 35(4), 1113–1121. http://ovidsp.ovid.com/ovidweb.cgi?T=JS&PAGE=reference&D=med4&NEWS=N&AN=10442879	EEG	89	44	45	noncompliance	0	44	0	0	0	experiment only	healthy / typical development	1	0	0	1	0	0	0	0	0	1	single session	0	1
Mash, C., Bornstein, M. H., & Arterberry, M. E. (2013). Brain dynamics in young infants' recognition of faces. *NeuroReport*, 24(7), 359–363. https://doi.org/10.1097/wnr.0b013e32835f6828	EEG	28	12	16	experimental errors,equipment failure	0	12	12	0	0	experiment	healthy	1	0	0	1	0	0	0	0	0	1	single session	0	1
Souweidane, M, et al.(1999). Brain Mapping in Sedated Infants and Young Children with Passive-Functional Magnetic Resonance Imaging. *Pediatric Neurosurgery*, 30(2), 86–92. https://doi.org/10.1159/000028768	fMRI	na	8	na	na	0	3	4	1	4	experiment	variious suspected and diagnosed neurological conditions	0	0	1	1	0	0	1	0	0	0	single session	1	0
S. Benavides-Varela R. Siugzdaite, et al. (2017). Brain regions and functional interactions supporting early word recognition in the face of input variability. *Proceedings of the National Academy of Sciences of the United States of America*, 114(29), 7588–7593. http://ovidsp.ovid.com/ovidweb.cgi?T=JS&PAGE=reference&D=med14&NEWS=N&AN=28674020	fNIRS	45	40	5	fussiness	0	40	0	0	0	experiment only	healthy / typical development	1	0	0	1	0	0	0	0	1	0	single session	0	1
Z. Radicevic M. Vujovic, L. J., & Sovilj, M. (2008). Comparative findings of voice and speech: language processing at an early ontogenetic age in quantitative EEG mapping. *Experimental Brain Research*, 184(4), 529–532. http://ovidsp.ovid.com/ovidweb.cgi?T=JS&PAGE=reference&D=med7&NEWS=N&AN=17934726	EEG	1	1	0		0	1	1	0	0	experiment	healthy	1	0	0	0	0	0	0	0	1	0	2 sessions	0	1
R. Slater, et al. (2006). Cortical pain responses in human infants. The Journal of Neuroscience : *The Official Journal of the Society for Neuroscience*, 26(14), 3662–3666. http://ovidsp.ovid.com/ovidweb.cgi?T=JS&PAGE=reference&D=med6&NEWS=N&AN=16597720	NIRS	na	18	na	na	0	18	18	0	0	experiment	healthy	1	1	0	1	0	0	1	0	0	0	single session	0	1
N. A. Folland B. E. Butler, J. E. P., & Trainor, L. J. (2015). Cortical representations sensitive to the number of perceived auditory objects emerge between 2 and 4 months of age: electrophysiological evidence. *Journal of Cognitive Neuroscience*, 27(5), 1060–1067. http://ovidsp.ovid.com/ovidweb.cgi?T=JS&PAGE=reference&D=med12&NEWS=N&AN=25436670	EEG	194	153	41	noncompliance, data artifacts	0	153	0	0	0	experiment only	healthy / typical development	1	0	0	1	0	0	0	0	1	1	single session	0	1
J. M. Stephen, et al. (2017). Development of Auditory Evoked Responses in Normally Developing Preschool Children and Children with Autism Spectrum Disorder. *Developmental Neuroscience*, 39(5), 430–441. http://ovidsp.ovid.com/ovidweb.cgi?T=JS&PAGE=reference&D=med14&NEWS=N&AN=28772264	MEG	na	64	na	na	0	na	26	na	38	experiment	mixed normal and autism spectrum disorder	0	1	0	1	0	0	1	0	1	0	single session	0	1
Sato, Y., Sogabe, Y., & Mazuka, R. (2010). Development of hemispheric specialization for lexical pitch-accent in Japanese infants. *Journal of Cognitive Neuroscience*, 22(11), 2503–2513. https://doi.org/10.1162/jocn.2009.21377	NIRS	50	40	10	technical problems, crying, experimental error	0	40	40	0	0	experiment	healthy	1	0	0	1	0	0	0	0	1	0	single session	0	1
Xie, W., Mallin, B. M., & Richards, J. E. (2018). Development of infant sustained attention and its relation to EEG oscillations: an EEG and cortical source analysis study. *Developmental Science*, 21(3), 1–16. https://doi.org/10.1111/desc.12562	EEG	68	36	32	noncompliance/fussy and Equipement/technical issues, excessive artifacts	0	24	36	12	0	experiment	healthy	1	0	0	1	0	0	0	0	0	1	single session	0	1
Marie, C., & Trainor, L. J. (2013). Development of simultaneous pitch encoding: Infants show a high voice superiority effect. *Cerebral Cortex*, 23(3), 660–669. https://doi.org/10.1093/cercor/bhs050	EEG	20	16	4	excessive movement and fussiness/noncompliance	0	20	20	0	0	experiment	healthy	1	0	0	1	0	0	0	0	1	0	single session	0	1
Arcand, C., et al. (2007). Development of visual texture segregation during the first year of life: A high-density electrophysiological study. *Experimental Brain Research*, 180(2), 263–272. https://doi.org/10.1007/s00221-007-0854-y	EEG	51	42	9	noncompliance, unusable data, movement artifacts	0	42	0	0	0	experiment only	healthy / typical development	1	0	0	1	1	0	1	0	0	1	single session	0	1
R. Ceschin, et al. (2015). Developmental synergy between thalamic structure and interhemispheric connectivity in the visual system of preterm infants. *NeuroImage*. Clinical, 8, 462–472. http://ovidsp.ovid.com/ovidweb.cgi?T=JS&PAGE=reference&D=med12&NEWS=N&AN=26106571	fMRI	38	38	na	na	22	16	0	0	0	experiment only	preterm, white-matter injury, healthy controls	0	0	1	1	0	0	0	0	0	1	single session	1	0
E. Nakato Y. Otsuka, S. K. M. K. Y., & Kakigi, R. (2011). Distinct differences in the pattern of hemodynamic response to happy and angry facial expressions in infants–a near-infrared spectroscopic study. *NeuroImage*, 54(2), 1600–1606. http://ovidsp.ovid.com/ovidweb.cgi?T=JS&PAGE=reference&D=med8&NEWS=N&AN=20850548	NIRS	21	12	9	body motion, lack of attention/ noncompliance, computer/technical error	0	12	12	0	0	experiment	healthy	1	0	0	1	0	0	0	0	0	1	single session	0	1
M. C. Moulson N. A. Fox, C. H. Z., & Nelson, C. A. (2009). Early adverse experiences and the neurobiology of facial emotion processing. *Developmental Psychology*, 45(1), 17–30. http://ovidsp.ovid.com/ovidweb.cgi?T=JS&PAGE=reference&D=med7&NEWS=N&AN=19209987	EEG & electro-oculogram	208	85	123	genetic syndromes, overt signs of fetal alcohol syndrome, technical errors	0	5 - 31months no break down 23.6 month median age				experiment	healthy	1	0	0	1	0	0	0	0	0	1	3 sessions	0	1
T. Grossmann, et al. (2008). Early cortical specialization for face-to-face communication in human infants. Proceedings. *Biological Sciences*, 275(1653), 2803–2811. http://ovidsp.ovid.com/ovidweb.cgi?T=JS&PAGE=reference&D=med7&NEWS=N&AN=18755668	EEG & fNRIS	44	24	20	unusable data	0	24	0	0	0	experiment only	healthy / typical development	1	0	0	0	1	0	0	0	0	1	single session	0	1
Mundy, P., Card, J., & Fox, N. (2000). EEG correlates of the development of infant joint attention skills. *Developmental Psychobiology*, 36(4), 325–338. https://doi.org/10.1002/(SICI)1098-2302(200005)36:4<325::AID-DEV7>3.0.CO;2-F	EEG	36	32	4	missing, compromised data	0	0	32	32	0	experiment	healthy	1	0	0	1	0	0	0	0	0	1	2 sessions	0	1
Watanabe, H., et al. (2013). Effect of auditory input on activations in infant diverse cortical regions during audiovisual processing. *Human Brain Mapping*, 34(3), 543–565. https://doi.org/10.1002/hbm.21453	NIRS	157	50	107	fussiness, motion/head movement artifacts, insufficient signal/data, hair obstruction	0	50	50	0	0	experiment	healthy	1	0	0	1	0	0	0	0	1	1	single session	0	1
Nishida, T., et al. (2008). Extrauterine environment affects the cortical responses to verbal stimulation in preterm infants. *Neuroscience Letters*, 443(1), 23–26. https://doi.org/10.1016/j.neulet.2008.07.035	NIOT	na	18	na	na	8	18	18	0	0	experiment	healthy 8 pre-term and 10 term	0	1	0	1	0	0	0	0	1	0	single session	0	1
Watanabe, H., Homae, F., Nakano, T., & Taga, G. (2008). Functional activation in diverse regions of the developing brain of human infants. *NeuroImage*, 43(2), 346–357. https://doi.org/10.1016/j.neuroimage.2008.07.014	NIRS	87	35	52	noncompliance/fussiness, head movement/motion artifacts, hair obstruction, insufficient data/signal	0	35	35	0	0	experiment	healthy	1	0	0	1	0	0	0	0	0	1	single session	0	1
Wilcox, T., Stubbs, J., Hirshkowitz, A., & Boas, D. A. (2012). Functional activation of the infant cortex during object processing. *NeuroImage*, 62(3), 1833–1840. https://doi.org/10.1016/j.neuroimage.2012.05.039	NIRS	148	111	37	prodedural issues, noncompliance/crying, technical signal, motion artifacts	0	111	111	0	0	experiment	healthy	1	0	0	1	0	0	0	0	0	1	single session	0	1
Homae, F., Watanabe, H., Nakano, T., & Taga, G. (2012). Functional development in the infant brain for auditory pitch processing. *Human Brain Mapping*, 33(3), 596–608. https://doi.org/10.1002/hbm.21236	fNIRS	84	46	38	unusable data or noncompliance, motion artifacts, hair obstruction	0	46	0	0	0	experiment only	healthy / typical development	0	1	0	1	0	0	0	0	1	0	single session	0	1
Haartsen, R., *et al.* (2019). Functional EEG connectivity in infants associates with later restricted and repetitive behaviours in autism; a replication study. *Translational Psychiatry*, 9(1). https://doi.org/10.1038/s41398-019-0380-2	EEG	143	101	42	unusable data or noncompliance	0	0	101	0	0	experiment only	patients with autism spectrum disorder and "low-risk" controls	1	0	0	0	1	0	0	0	1	1	single session	1	0
Isobe, K., et al. (2001). Functional imaging of the brain in sedated newborn infants using near infrared topography during passive knee movement. *Neuroscience Letters*, 299(3), 221–224. https://doi.org/10.1016/S0304-3940(01)01518-X	fNIRS	7	7	na	na	5	2	0	0	0	experiment only	healthy / typical development and healthy / preterm development	0	0	1	1	0	0	0	1	0	0	single session	0	1
T. Kusaka, et al. (2011). Functional lateralization of sensorimotor cortex in infants measured using multichannel near-infrared spectroscopy. *Pediatric Research*, 69(5), 430–435. http://ovidsp.ovid.com/ovidweb.cgi?T=JS&PAGE=reference&D=med8&NEWS=N&AN=21283052	MNIRS	na	10	na	na	2	10	10	0	0	Clinical	mixed neurological conditions, preterm, term	0	0	1	1	0	0	1	1	0	0	single session	1	0
A. P. Born, et al. (2000). Functional magnetic resonance imaging of the normal and abnormal visual system in early life. *Neuropediatrics*, 31(1), 24–32. http://ovidsp.ovid.com/ovidweb.cgi?T=JS&PAGE=reference&D=med4&NEWS=N&AN=10774992	fMRI	37	30	7	unusable data	5	17	0	1	3	experiment only	preterm, visual impairment, and healthy controls	0	1	1	1	1	0	0	0	0	1	1 or 2	0	1
Heep, A., et al. (2009). Functional Magnetic Resonance Imaging of the Sensorimotor System in Preterm Infants. *Pediatrics*, 123(1), 294–300. https://doi.org/10.1542/peds.2007-3475	fMRI	11	8	3	unusable or incomplete data, movement artifacts	8	0	0	0	0	experiment only	healthy / preterm birth	0	0	1	1	1	1	1	1	0	0	single session	0	1
Ives-Deliperi, V. L., & Butler, J. T. (2015). Functional Mapping in Pediatric Epilepsy Surgical Candidates: Functional Magnetic Resonance Imaging Under Sedation With Chloral Hydrate. *Pediatric Neurology*, 53(6), 478–484. http://ovidsp.ovid.com/ovidweb.cgi?T=JS&PAGE=reference&D=med12&NEWS=N&AN=26431897	fMRI	24	24	na	na	0	0	0	3	21	routine presurgical scanning	epilepsy patients	0	0	1	1	1	0	0	1	1	0	single session	1	0
Bembich, S., et al. (2013). Functional neuroimaging of breastfeeding analgesia by multichannel near-infrared spectroscopy. *Neonatology*, 104(4), 255–259. https://doi.org/10.1159/000353386	fNIRS	30	30	na	na	0	30	0	0	0	experiment only	healthy / typical development	1	0	0	0	1	0	1	0	0	0	single session	1	1
G. Dehaene-Lambertz, et al. (2006). Functional organization of perisylvian activation during presentation of sentences in preverbal infants. *Proceedings of the National Academy of Sciences of the United States of America*, 103(38), 14240–14245. http://ovidsp.ovid.com/ovidweb.cgi?T=JS&PAGE=reference&D=med6&NEWS=N&AN=16968771	fMRI	11	11	na	na	0	11	0	0	0	experiment only	healthy / typical development	1	0	0	1	0	0	0	0	1	0	single session	0	1
Davis, A. E., & Wada, J. A. (1977). Hemispheric asymmetries in human infants: spectral analysis of flash and click evoked potentials. *Brain and Language*, 4(1), 23–31. http://ovidsp.ovid.com/ovidweb.cgi?T=JS&PAGE=reference&D=med1&NEWS=N&AN=832104	EEG	16	16	na	na	0	16	0	0	0	experiment only	healthy / typical development	1	0	0	1	0	0	0	0	1	1	single session	0	1
Karen, T., et al. (2008). Hemodynamic response to visual stimulation in newborn infants using functional near-infrared spectroscopy. *Human Brain Mapping*, 29(4), 453–460. https://doi.org/10.1002/hbm.20411	fNIRS	20	20	na	na	0	20	0	0	0	experiment only	healthy / typical development	0	1	0	1	0	0	0	0	0	1	single session	0	1
S. M. Liao, et al.(2012). High-density diffuse optical tomography of term infant visual cortex in the nursery. *Journal of Biomedical Optics*, 17(8), 81414. http://ovidsp.ovid.com/ovidweb.cgi?T=JS&PAGE=reference&D=med9&NEWS=N&AN=23224175	HD-DOT	11	7	4	noise and motion artifacts	0	11	11	0	0	experiment	healthy	1	1	0	1	0	0	0	0	0	1	single session	0	1
Piazza, C., et al. (2016). ICA-derived cortical responses indexing rapid multi-feature auditory processing in six-month-old infants. *NeuroImage*, 133, 75–87. https://doi.org/10.1016/j.neuroimage.2016.02.060	EEG	39	34	5	insufficient good EEG data	0	34	34	0	0	experiment	healthy	1	0	0	1	0	0	0	0	1	0	single session	0	1
H. Bortfeld, E. F., & Boas, D. A. (2009). Identifying cortical lateralization of speech processing in infants using near-infrared spectroscopy. *Developmental Neuropsychology*, 34(1), 52–65. http://ovidsp.ovid.com/ovidweb.cgi?T=JS&PAGE=reference&D=med7&NEWS=N&AN=19142766	fNIRS	28	21	7	unusable data, hair obstruction, motion	0	21	0	0	0	experiment only	healthy / typical development	1	0	0	1	1	0	0	0	1	1	single session	0	1
G. D. Reynolds, M. L. C., & Richards, J. E. (2010). Infant attention and visual preferences: converging evidence from behavior, event-related potentials, and cortical source localization. *Developmental Psychology*, 46(4), 886–904. http://ovidsp.ovid.com/ovidweb.cgi?T=JS&PAGE=reference&D=med8&NEWS=N&AN=20604609	EEG	71	47	24	fussiness, artifacts,inattentiveness,technical	0	47	47	0	0	experiment	healthy	1	0	0	1	0	0	0	0	0	1	single session	0	1
Farroni, T., et al. (2013). Infant cortex responds to other humans from shortly after birth. *Scientific Reports*, 3, 3–7. https://doi.org/10.1038/srep02851	fNIRS	17	15	2	noncompliance, technical issues, noissy data	0	15	0	0	0	experiment only	healthy / typical development	1	0	0	0	1	0	0	0	1	1	single session	0	1
T. Imada, et al. (2006). Infant speech perception activates Broca's area: a developmental magnetoencephalography study. *Neuroreport*, 17(10), 957–962. http://ovidsp.ovid.com/ovidweb.cgi?T=JS&PAGE=reference&D=med6&NEWS=N&AN=16791084	MEG	43	43	na	na	0	43	0	0	0	experiment only	healthy / typical development	na	na	na	1	0	0	0	0	1	0	single session	0	1
Dawson, G., Panagiotides, H., Klinger, L. G., & Spieker, S. (1997). Infants of depressed and nondepressed mothers exhibit differences in frontal brain electrical activity during the expression of negative emotions. *Developmental Psychology*, 33(4), 650–656. https://doi.org/10.1037/0012-1649.33.4.650	EEG	34	34	na	na	0	0	34	0	0	experiment only	healthy / typical development	1	0	0	0	1	0	0	0	0	1	single session	0	1
Blasi, A., et al. (2007). Investigation of depth dependent changes in cerebral haemodynamics during face perception in infants. *Physics in Medicine and Biology*, 52(23), 6849–6864. https://doi.org/10.1088/0031-9155/52/23/005	fNIRS	38	12	26	excessive head movement, noncompliance	0	12	0	0	0	experiment only	healthy / typical development	1	0	0	1	0	0	0	0	0	1	single session	0	1
Richards, J. E. (2005). Localizing cortical sources of event-related potentials in infants' covert orienting. *Developmental Science*, 8(3), 255–278. http://ovidsp.ovid.com/ovidweb.cgi?T=JS&PAGE=reference&D=med6&NEWS=N&AN=15819757	HD-EEG	33	22	11	fussy,sleepy or insufficiennt data, equipmemt problems	0	22	22	0	0	experiment	healthy	1	0	0	0	1	1	0	0	0	1	single session	0	1
Verriotis, M., Fabrizi, L., Lee, A., Cooper, R. J., Fitzgerald, M., & Meek, J. (2016). Mapping cortical responses to somatosensory stimuli in human infants with simultaneous near-infrared spectroscopy and event-related potential recording. *ENeuro*, 3(2), 663–673. https://doi.org/10.1523/ENEURO.0026-16.2016	NIRS & EEG	36	30	6	limited time 6 did not receive EEG, technical/poor data/signal quality	0	30	30	0	0	experiment	healthy term infants	0	1	0	1	0	0	1	0	0	0	single session	0	1
Uchida-Ota, M., et al. (2019). Maternal speech shapes the cerebral frontotemporal network in neonates: A hemodynamic functional connectivity study. *Developmental Cognitive Neuroscience*, 39(January), 100701. https://doi.org/10.1016/j.dcn.2019.100701	NIRS	na	34	na	na	0	37	37	0	0	experiment	healthy	0	1	0	1	0	0	0	0	1	0	single session	0	1
E. Pihko P. Nevalainen, J. S. Y. O., & Lauronen, L. (2009). Maturation of somatosensory cortical processing from birth to adulthood revealed by magnetoencephalography. Clinical Neurophysiology : *Official Journal of the International Federation of Clinical Neurophysiology*, 120(8), 1552–1561. http://ovidsp.ovid.com/ovidweb.cgi?T=JS&PAGE=reference&D=med7&NEWS=N&AN=19560400	MEG	57	51	6	na	0	28	36	8	20	experiment	healthy	1	1	0	1	0	0	1	0	0	0	single session	0	1

Imafuku, M., Hakuno, Y., Uchida-Ota, M., Yamamoto, J. ichi, & Minagawa, Y. (2014). “Mom called me!” Behavioral and prefrontal responses of infants to self-names spoken by their mothers. *NeuroImage*, 103, 476–484. https://doi.org/10.1016/j.neuroimage.2014.08.034	fNIRS	34	27	7	fussiness or technical issue	0	27	0	0	0	experiment only	healthy / typical development	1	0	0	0	1	0	0	0	1	1	single session	0	1
M. Biallas, et al. (2012). Multimodal recording of brain activity in term newborns during photic stimulation by near-infrared spectroscopy and electroencephalography. *Journal of Biomedical Optics*, 17(8), 86011. http://ovidsp.ovid.com/ovidweb.cgi?T=JS&PAGE=reference&D=med9&NEWS=N&AN=23224198	fNIRS + EEG	14	14	na	na	0	14	0	0	0	experiment only	healthy / typical development	0	1	0	1	0	0	0	0	0	1	single session	0	1
Liao, S. M., et al. (2010). Neonatal hemodynamic response to visual cortex activity: high-density near-infrared spectroscopy study. *Journal of Biomedical Optics*, 15(2), 26010. https://doi.org/10.1117/1.3369809	HD-NIRS	11	11	0	na	0	11	11	0	0	experiment	healthy	1	0	0	1	0	0	0	0	0	1	single session	0	1
Shultz, S., Vouloumanos, A., Bennett, R. H., & Pelphrey, K. (2014). Neural specialization for speech in the first months of life. *Developmental Science*, 17(5), 766–774. https://doi.org/10.1111/desc.12151	fMRI	38	24	14	excessive motion	0	24	24	0	0	experimnt	healthy	0	1	0	1	0	0	0	0	1	0	2 sessions	0	1
Siddiqui, M. F., et al. (2017). Non-invasive measurement of a metabolic marker of infant brain function. *Scientific Reports*, 7(1), 1–6. https://doi.org/10.1038/s41598-017-01394-z	NIRS	33	24	9	technical issues, noncompliance, inverted response to experimental stimuli	0	24	24	0	0	experiment	healthy	1	0	0	1	0	0	0	0	1	1	single session	0	1
Kusaka, T., et al. (2004). Noninvasive optical imaging in the visual cortex in young infants. *Human Brain Mapping*, 22(2), 122–132. https://doi.org/10.1002/hbm.20020	fNIRS	10	10	na	na	5	0	0	0	5	experiment only	various conditions / preterm birth and healthy adult controls	0	1	0	1	0	0	0	0	0	1	single session	0	1
Minagawa-Kawai, Y., et al. (2011). Optical brain imaging reveals general auditory and language-specific processing in early infant development. *Cerebral Cortex*, 21(2), 254–261. https://doi.org/10.1093/cercor/bhq082	NIRS	30	12	18	head movement/ motion artifacts, fussiness/noncompliance, hair obstruction	0	12	12	0	0	experiment	healthy	1	0	0	1	0	0	0	0	1	0	single session	0	1
Ogg, R. J., et al. (2009). Passive range of motion functional magnetic resonance imaging localizing sensorimotor cortex in sedated children: Clinical article. *Journal of Neurosurgery: Pediatrics*, 4(4), 317–322. https://doi.org/10.3171/2009.4.PEDS08402	16-BOLD fMRI & 9-ECOG	na	16	na	na	0	0	4	4	12	Clinical	Patients with neurological impairments, lesons	1	0	1	1	0	0	1	1	0	0	single session	1	0
Routier, L., et al.(2017). Plasticity of neonatal neuronal networks in very premature infants: Source localization of temporal theta activity, the first endogenous neural biomarker, in temporoparietal areas. *Human Brain Mapping*, 38(5), 2345–2358. https://doi.org/10.1002/hbm.23521	LD/HD-EEG	na	28	na	na	28	28	28	0	0	experiment	healthy	0	1	0	1	0	0	0	0	1	0	single session	0	1
Gou, Z., Choudhury, N., & Benasich, A. A. (2011). Resting frontal gamma power at 16, 24 and 36 months predicts individual differences in language and cognition at 4 and 5 years. *Behavioural Brain Research*, 220(2), 263–270. https://doi.org/10.1016/j.bbr.2011.01.048	EEG	46	40	6	incomplete dataset	0	0	40	0	0	experiment only	healthy / typical development	1	0	0	1	0	0	0	0	1	0	3+ sessions	0	1
Lloyd-Fox, S., Blasi, A., Everdell, N., Elwell, C. E., & Johnson, M. H. (2011). Selective cortical mapping of biological motion processing in young infants. *Journal of Cognitive Neuroscience*, 23(9), 2521–2532. https://doi.org/10.1162/jocn.2010.21598	NIRS	24	13	11	technical/thick hair, noncompliance	0	13	13	0	0	experiment	healthy	1	0	0	1	0	0	0	0	0	1	single session	0	1
Taga, G., & Asakawa, K. (2007). Selectivity and localization of cortical response to auditory and visual stimulation in awake infants aged 2 to 4 months. *NeuroImage*, 36(4), 1246–1252. http://ovidsp.ovid.com/ovidweb.cgi?T=JS&PAGE=reference&D=med6&NEWS=N&AN=17524672	NIOT	45	15	30	head movement/motion artifacts, noncompliance, technical/hair obstruction and electrode placement	0	15	15	0	0	experiment	healthy	1	0	0	1	0	0	0	0	1	1	single session	0	1
Kobayashi, M., Otsuka, Y., Kanazawa, S., Yamaguchi, M. K., & Kakigi, R. (2012). Size-invariant representation of face in infant brain: An fNIRS-adaptation study. *NeuroReport*, 23(17), 984–988. https://doi.org/10.1097/WNR.0b013e32835a4b86	fNIRS	26	15	11	noncompliance or incomplete data, motion artifacts, hair interference	0	15	0	0	0	experiment only	healthy / typical development	0	1	0	0	1	0	0	0	0	1	single session	0	1
Arichi, T., et al. (2010). Somatosensory cortical activation identified by functional MRI in preterm and term infants. *NeuroImage*, 49(3), 2063–2071. https://doi.org/10.1016/j.neuroimage.2009.10.038	fMRI	40	40	na	na	13	27	0	0	0	experiment only	healthy / typical development	0	1	1	1	1	1	1	1	0	0	single session	0	1
Dall’Orso, S., et al. (2018). Somatotopic mapping of the developing sensorimotor cortex in the preterm human brain. *Cerebral Cortex*, 28(7), 2507–2515. https://doi.org/10.1093/cercor/bhy050	fMRI	35	35	na	na	35	0	0	0	0	experiment only	healthy / preterm birth, some lesions	0	1	0	1	1	1	1	1	0	0	single session	0	1
Bembich, S., et al. (2016). The cortical response to a noxious procedure changes over time in preterm infants. *Pain*, 157(9), 1979–1987. https://doi.org/10.1097/j.pain.0000000000000605	fNIRS	16	16	na	na	16	0	0	0	0	experiment + routine blood draws	healthy / preterm birth	1	0	0	0	1	0	1	0	0	0	3+ sessions	1	0
A. M. Boldin, R. G., & Emberson, L. L. (2018). The emergence of top-down, sensory prediction during learning in infancy: A comparison of full-term and preterm infants. *Developmental Psychobiology*, 60(5), 544–556. http://ovidsp.ovid.com/ovidweb.cgi?T=JS&PAGE=reference&D=med15&NEWS=N&AN=29687654	fNIRS	100	79	21	noncompliance (20) + electrode issue (1), missing signal/data, hair obstruction	43	36	0	0	0	experiment only	healthy / typical development	1	0	0	1	0	0	0	0	1	1	single session	0	1
Nystrom, P. (2008). The infant mirror neuron system studied with high density EEG. *Social Neuroscience*, 3(4), 334–347. http://ovidsp.ovid.com/ovidweb.cgi?T=JS&PAGE=reference&D=med7&NEWS=N&AN=18979389	HD-EEG	57	34	23	noncompliance/fussiness & technical issues	0	19	19	0	15	experiment	healthy	1	0	0	1	0	0	0	0	0	1	single session	0	1
Reid, V. M., et al. (2009). The Neural Correlates of Infant and Adult Goal Prediction: Evidence for Semantic Processing Systems. *Developmental Psychology*, 45(3), 620–629. https://doi.org/10.1037/a0015209	EEG	88	42	46	fussiness, failure to meet data inclusion criterior	0	27	27	0	15	experiment	healthy	1	0	0	1	0	0	0	0	0	1	single session	0	1
Gervain, J., Werker, J. F., Black, A., & Geffen, M. N. (2016). The neural correlates of processing scale-invariant environmental sounds at birth. *NeuroImage*, 133, 144–150. https://doi.org/10.1016/j.neuroimage.2016.03.001	fNIRS	30	22	8	noncompliance or thick hair, data quality, motion artifacts	0	22	0	0	0	experiment only	healthy / typical development	0	1	0	1	0	0	0	0	1	0	single session	1	0
Altvater-Mackensen, N., & Grossmann, T. (2016). The role of left inferior frontal cortex during audiovisual speech perception in infants. *NeuroImage*, 133, 14–20. https://doi.org/10.1016/j.neuroimage.2016.02.061	fNIRS	30	24	6	noncompliance	0	24	0	0	0	experiment only	healthy / typical development	1	0	0	1	0	0	0	0	1	1	single session	0	1
Shen, G., Weiss, S. M., Meltzoff, A. N., & Marshall, P. J. (2018). The somatosensory mismatch negativity as a window into body representations in infancy. *International Journal of Psychophysiology*, 134(October), 144–150. https://doi.org/10.1016/j.ijpsycho.2018.10.013	EEG	33	31	2	artifacts, excessive movement, data/signal artifacts,	0	31	31	0	0	experiment	healthy	1	0	0	1	0	0	1	0	0	0	single session	0	1
Ortiz-Mantilla, S., Hämäläinen, J. A., & Benasich, A. A. (2012). Time course of ERP generators to syllables in infants: A source localization study using age-appropriate brain templates. *NeuroImage*, 59(4), 3275–3287. https://doi.org/10.1016/j.neuroimage.2011.11.048	EEG/ERP & MRI	42	28	14	high noise and medical condition	0	28	28	0	0	experiment	healthy	1	1	0	1	0	0	0	0	1	0	single session	0	1
Isler, J. R., et al. (2012). Toward an electrocortical biomarker of cognition for newborn infants. *Developmental Science*, 15(2), 260–271. https://doi.org/10.1111/j.1467-7687.2011.01122.x	EEG	32	21	11	excessive head movement	0	21	0	0	0	experiment only	healthy / typical development	0	1	0	1	0	0	0	0	1	0	single session	0	1
Emberson, L. L., et al. (2017). Using fNIRS to examine occipital and temporal responses to stimulus repetition in young infants: Evidence of selective frontal cortex involvement. *Developmental Cognitive Neuroscience*, 23, 26–38. https://doi.org/10.1016/j.dcn.2016.11.002	fNIRS	26	18	8	noncompliance/fussiness, excessive noise data, hair obstruction	0	18	0	0	0	experiment only	healthy / typical development	1	0	0	1	0	0	0	0	1	1	single session	0	1
Bernal, B., & Altman, N. (2004). Visual functional magnetic resonance imaging in patients with Sturge-Weber syndrome. *Pediatric Neurology*, 31(1), 15-Sep. http://ovidsp.ovid.com/ovidweb.cgi?T=JS&PAGE=reference&D=med5&NEWS=N&AN=15246485	fMRI	17	17	na	na	0	10	0	0	7	experiment only	patients with sturge-weber syndrome, and healthy controls	0	0	1	1	0	0	0	0	0	1	single session	0	1
Carver, L. J., & Vaccaro, B. G. (2007). 12-month-old infants allocate increased neural resources to stimuli associated with negative adult emotion. *Developmental Psychology*, 43(1), 54–69. http://ovidsp.ovid.com/ovidweb.cgi?T=JS&PAGE=reference&D=med6&NEWS=N&AN=17201508	EEG	55	34	21	noncompliance, motion artifacts	0	34	0	0	0	experiment only	healthy/typical development	1	0	0	1	1	0	0	0	1	1	single session	0	1
A. Ritterband-Rosenbaum, et al. (2017). A critical period of corticomuscular and EMG–EMG coherence detection in healthy infants aged 9–25 weeks. *The Journal of Physiology*, 595(8), 2699–2713. https://www.proquest.com/scholarly-journals/critical-period-corticomuscular-emg-coherence/docview/1870294751/se-2	EEG	61	59	2	unusable data	0	0	59	0	0	experiment only	healthy/typical development	1	0	0	0	1	0	0	1	0	0	single session	0	1
A. Donadio K. Whitehead, et al. (2018). A novel sensor design for accurate measurement of facial somatosensation in pre-term infants. *PLoS One*, 13(11). https://www.proquest.com/scholarly-journals/novel-sensor-design-accurate-measurement-facial/docview/2134282466/se-2	EEG	7	7	0	na	7	0	0	0	0	experiment only	healthy/preterm	0	1	0	0	1	0	1	0	0	0	single session	0	1
Yamada, H., et al. (1997). A rapid brain metabolic change in infants detected by fMRI. *NeuroReport*, 8(17), 3775–3778. https://doi.org/10.1097/00001756-199712010-00024	fMRI	15	15	0	na	0	0	15	0	0	experiment only	possible brain damage	0	0	1	1	0	0	0	0	0	1	single session	0	1
E. Parise, A. D. F., & Striano, T. (2010). “Did You Call Me?” 5-Month-Old Infants Own Name Guides Their Attention. *PLoS One*, 5(12). https://www.proquest.com/scholarly-journals/did-you-call-me-5-month-old-infants-own-name/docview/1318939962/se-2	EEG	55	30	25	noncompliance	0	30	0	0	0	experiment only	healthy/typical development	1	0	0	1	0	0	0	0	1	1	single session	0	1
H. Watanabe, F. H., & Taga, G. (2012). Activation and deactivation in response to visual stimulation in the occipital cortex of 6-month-old human infants. *Developmental Psychobiology*, 54(1), 15-Jan. http://ovidsp.ovid.com/ovidweb.cgi?T=JS&PAGE=reference&D=med9&NEWS=N&AN=21594872	fNIRS	87	35	52	noncompliance, motion artifacts, hair interference	0	35	0	0	0	experiment only	healthy/typical development	1	0	0	1	0	0	0	0	1	1	single session	0	1
L. J. Carver G. Dawson, et al. (2003). Age-related differences in neural correlates of face recognition during the toddler and preschool years. *Developmental Psychobiology*, 42(2), 148–159. http://ovidsp.ovid.com/ovidweb.cgi?T=JS&PAGE=reference&D=med5&NEWS=N&AN=12555279	EEG	75	42	33	motion artifacts, noncompliance	0	0	0	42	0	experiment only	healthy/typical development	1	0	0	1	0	0	0	0	0	1	single session	0	1
Posikera, I. N., & Stronganova, T. A. (1984). An electroencephalographic analysis of the functional development of the cortical regions of the brain in children during the first month of life. *Soviet Psychology*, 22(3), 47–60. https://www.proquest.com/scholarly-journals/electroencephalographic-analysis-functional/docview/616973028/se-2?accountid=14521	EEG	na	50	na	na	0	50	0	0	0	experiment only	healthy/typical development	1	0	0	1	0	0	0	0	0	1	single session	0	1
S. Lloyd-Fox B. Szeplaki-Kollod, J. Y., & Csibra, G. (2015). Are you talking to me? Neural activations in 6-month-old infants in response to being addressed during natural interactions. Cortex; *a Journal Devoted to the Study of the Nervous System and Behavior*, 70, 35–48. http://ovidsp.ovid.com/ovidweb.cgi?T=JS&PAGE=reference&D=med12&NEWS=N&AN=25891796	fNIRS	52	24	28	noncompliance, distraction, failure to attend, device malfunction	0	24	0	0	0	experiment only	healthy/typical development	1	0	0	1	0	0	0	0	1	1	single-session	0	1
Y. Minagawa-Kawai A. Cristià, I. V. D. C., & Dupoux, E. (2011). Assessing signal-driven mechanisms in neonates: Brain responses to temporally and spectrally different sounds. *Frontiers in Psychology*, 2, 12. https://www.proquest.com/scholarly-journals/assessing-signal-driven-mechanisms-neonates-brain/docview/2011633341/se-2	fNIRS	38	29	9	motion artifacts, discomfort	0	29	0	0	0	experiment only	healthy/typical development	1	0	0	1	0	0	0	0	1	0	single-session	0	1
C. T. Ellis L. J. Skalaban, T. S. Y., & Turk-Browne, N. B. (2021). Attention recruits frontal cortex in human infants. *Proceedings of the National Academy of Sciences of the United States of America*, 118(12). http://ovidsp.ovid.com/ovidweb.cgi?T=JS&PAGE=reference&D=medl&NEWS=N&AN=33727420	fMRI	31	20	11	lack of attention, motion artifacts	0	20	0	0	0	experiment only	healthy/typical development	1	0	0	1	0	0	0	0	0	1	single-session/two sessions	0	1
J. P. McCleery N. Akshoomoff, K. R. D., & Carver, L. J. (2009). Atypical face versus object processing and hemispheric asymmetries in 10-month-old infants at risk for autism. *Biological Psychiatry*, 66(10), 950–957. http://ovidsp.ovid.com/ovidweb.cgi?T=JS&PAGE=reference&D=med7&NEWS=N&AN=19765688	EEG	40	40	na	na	0	40	0	0	0	experiment only	Risk of developing ASD	1	0	0	1	0	0	0	0	0	1	single-session	0	1
E. Jansson-Verkasalo, et al. (2010). Atypical perceptual narrowing in prematurely born infants is associated with compromised language acquisition at 2 years of age. *BMC Neuroscience*, 11, 88. http://ovidsp.ovid.com/ovidweb.cgi?T=JS&PAGE=reference&D=med8&NEWS=N&AN=20673357	EEG	24	24	na	na	11	13	0	0	0	experiment only	Possible language deficit/healthy	1	0	0	1	0	0	0	0	1	0	single-session	0	1
A. Blasi, et al. (2015). Atypical processing of voice sounds in infants at risk for autism spectrum disorder. Cortex; *a Journal Devoted to the Study of the Nervous System and Behavior*, 71, 122–133. http://ovidsp.ovid.com/ovidweb.cgi?T=JS&PAGE=reference&D=med12&NEWS=N&AN=26200892	fMRI	35	33	2	incomplete datasets/ASD diagnosis	0	33	0	0	0	experiment only	Risk of developing ASD	0	1	0	1	0	0	0	0	1	0	single-session	0	1
Molfese, D. L. (1990). Auditory evoked responses recorded from 16-month-old human infants to words they did and did not know. *Brain and Language*, 38(3), 345–363. http://ovidsp.ovid.com/ovidweb.cgi?T=JS&PAGE=reference&D=med3&NEWS=N&AN=2346876	EEG	18	18	na	na	0	0	0	18	0	experiment only	healthy/typical development	1	0	0	1	0	0	0	0	1	0	single-session	0	1
X. Mai, et al. (2012). Auditory recognition memory in 2-month-old infants as assessed by event-related potentials. *Developmental Neuropsychology*, 37(5), 400–414. http://ovidsp.ovid.com/ovidweb.cgi?T=JS&PAGE=reference&D=med9&NEWS=N&AN=22799760	EEG	49	26	23	na	0	26	0	0	0	experiment only	healthy/typical development	1	0	0	1	0	0	0	0	1	0	single-session	0	1
Di Francesco, M. W., Robertson, S. A., Karunanayaka, P., & Holland, S. K. (2013). BOLD fMRI in infants under sedation: Comparing the impact of pentobarbital and propofol on auditory and language activation. *Journal of Magnetic Resonance Imaging*, 38(5), 1184–1195. https://doi.org/10.1002/jmri.24082	fMRI	20	20	na	na	0	0	20	0	0	experiment only	healthy/typical development	0	0	1	1	0	0	0	0	1	0	single-session	0	1
T. Grossmann, S. L.-F., & Johnson, M. H. (2013). Brain responses reveal young infants’ sensitivity to when a social partner follows their gaze. *Developmental Cognitive Neuroscience*, 6, 155–161. https://www.proquest.com/scholarly-journals/brain-responses-reveal-young-infants-sensitivity/docview/1531962761/se-2	fNIRS	17	12	5	motion artifacts	0	12	0	0	0	experiment only	healthy/typical development	1	0	0	1	0	0	0	0	0	1	single-session	0	1
R. Di Lorenzo A. Blasi, C. J. C. van den B. R. van R., & Kemner, C. (2019). Brain responses to faces and facial expressions in 5-month-olds: An fNIRS study. *Frontiers in Psychology*, 10, 8. https://www.proquest.com/scholarly-journals/brain-responses-faces-facial-expressions-5-month/docview/2434539258/se-2?accountid=14521	fNIRS	19	17	2	did not view all trials	0	17	0	0	0	experiment only	healthy/typical development	1	0	0	1	0	0	0	0	0	1	single-session	0	1
M. M. Swingler, M. A. S., & Carver, L. J. (2010). Brain-behavior correlations: relationships between mother-stranger face processing and infants’ behavioral responses to a separation from mother. *Developmental Psychology*, 46(3), 669–680. http://ovidsp.ovid.com/ovidweb.cgi?T=JS&PAGE=reference&D=med8&NEWS=N&AN=20438178	EEG	55	30	25	noncompliance, artifacts, error	0	30	0	0	0	experiment only	healthy/typical development	1	0	0	1	0	0	0	0	0	1	single-session	0	1
M. Shibata, et al. (2012). Broad cortical activation in response to tactile stimulation in newborns. *Neuroreport*, 23(6), 373–377. http://ovidsp.ovid.com/ovidweb.cgi?T=JS&PAGE=reference&D=med9&NEWS=N&AN=22395654	fNIRS	10	10	na	na	0	10	0	0	0	experiment only	healthy/typical development	1	0	0	1	1	0	1	0	1	1	single-session	0	1
E. Geangu, et al. (2015). By the sound of it. An ERP investigation of human action sound processing in 7-month-old infants. *Developmental Cognitive Neuroscience*, 12, 134–144. http://ovidsp.ovid.com/ovidweb.cgi?T=JS&PAGE=reference&D=med12&NEWS=N&AN=25732377	EEG	23	15	8	noncompliance, artifacts, device error	0	15	0	0	0	experiment only	healthy/typical development	1	0	0	1	0	0	0	0	1	0	single-session	0	1
H. Sato, et al. (2012). Cerebral hemodynamics in newborn infants exposed to speech sounds: a whole-head optical topography study. *Human Brain Mapping*, 33(9), 2092–2103. http://ovidsp.ovid.com/ovidweb.cgi?T=JS&PAGE=reference&D=med9&NEWS=N&AN=21714036	fNIRS	17	17	na	na	0	17	0	0	0	experiment only	healthy/typical development	0	1	0	1	0	0	0	0	1	0	single-session	0	1
N. Naoi, et al. (2012). Cerebral responses to infant-directed speech and the effect of talker familiarity. *NeuroImage*, 59(2), 1735–1744. http://ovidsp.ovid.com/ovidweb.cgi?T=JS&PAGE=reference&D=med9&NEWS=N&AN=21867764	fNIRS	105	48	57	noncompliance, motion artifacts, hair obstruction	0	33	15	0	0	experiment only	healthy/typical development	1	0	0	1	0	0	0	0	1	0	two-session	0	1
Bell, M. A., & Wolfe, C. D. (2007). Changes in brain functioning from infancy to early childhood: Evidence from EEG power and coherence during working memory tasks. Developmental *Neuropsychology*, 31(1), 21–38. https://www.proquest.com/scholarly-journals/changes-brain-functioning-infancy-early-childhood/docview/621648565/se-2?accountid=14521	EEG	50	50	na	na	0	50	0	0	0	experiment only	healthy/typical development	1	0	0	0	1	1	0	0	0	1	two-session	0	1
B. Z. Karmel, et al. (1977). Correlation of infants’ brain and behavior response to temporal changes in visual stimulation. *Psychophysiology*, 14(2), 134–142. http://ovidsp.ovid.com/ovidweb.cgi?T=JS&PAGE=reference&D=med1&NEWS=N&AN=847064	EEG	32	14	18	noncompliance, sleep	0	14	0	0	0	experiment only	healthy/typical development	1	0	0	1	0	0	0	0	0	1	two-session	0	1
S. Lloyd-Fox, et al. (2015). Cortical activation to action perception is associated with action production abilities in young infants. *Cerebral Cortex*, 25(2), 289–297. https://www.proquest.com/scholarly-journals/cortical-activation-action-perception-is/docview/1655757767/se-2	fNIRS	36	24	12	artifacts, noncompliance	0	24	0	0	0	experiment only	healthy/typical development	1	0	0	1	0	0	0	0	0	1	single-session	0	1

van Leeuwen P., et al. (2007). Cortical categorization failure in 2-month-old infants at risk for dyslexia. *Neuroreport*, 18(9), 857–861. http://ovidsp.ovid.com/ovidweb.cgi?T=JS&PAGE=reference&D=med6&NEWS=N&AN=17515790	EEG	50	50	na	na	0	50	0	0	0	experiment only	Genetic risk of dyslexia	0	1	0	1	0	0	0	0	1	0	single-session	0	1
G. Csibra L. A. Tucker, A. V., & Johnson, M. H. (2000). Cortical development and saccade planning: the ontogeny of the spike potential. *Neuroreport*, 11(5), 1069–1073. http://ovidsp.ovid.com/ovidweb.cgi?T=JS&PAGE=reference&D=med4&NEWS=N&AN=10790884	EEG	26	10	16	motion artifcats, lack of trial completions	0	0	10	0	0	experiment only	healthy/typical development	1	0	0	1	0	0	0	0	1	1	single-session	0	1
K. A. Gordon, D. D. E. W., & Papsin, B. C. (2010). Cortical function in children receiving bilateral cochlear implants simultaneously or after a period of interimplant delay. *Otology & Neurotology : Official Publication of the American Otological Society, American Neurotology Society [and] European Academy of Otology and Neurotology*, 31(8), 1293–1299. http://ovidsp.ovid.com/ovidweb.cgi?T=JS&PAGE=reference&D=med8&NEWS=N&AN=20634775	EEG	8	8	0	na	0	4	4	0	0	experiment only	deaf with cochlear implant	1	0	0	1	0	0	0	0	1	0	single-session	0	1
L. J. Trainor, K. L., & Bosnyak, D. J. (2011). Cortical plasticity in 4-month-old infants: specific effects of experience with musical timbres. *Brain Topography*, 24(4), 192–203. http://ovidsp.ovid.com/ovidweb.cgi?T=JS&PAGE=reference&D=med8&NEWS=N&AN=21445665	EEG	38	25	13	artifacts	0	25	0	0	0	experiment only	healthy/typical development	1	0	0	1	0	0	0	0	1	0	single-session	0	1
Lloyd-Fox, S., et al. (2018). Cortical responses before 6 months of life associate with later autism. *European Journal of Neuroscience*, 47(6), 736–749. https://doi.org/10.1111/ejn.13757	fNIRS	36	36	na	na	0	36	0	0	0	experiment only	Risk of developing ASD	1	0	0	1	0	0	0	0	1	1	single-session	0	1
G. P. Novak D. Kurtzberg, J. A. K., & H. G. Vaughan, Jr. (1989). Cortical responses to speech sounds and their formants in normal infants: maturational sequence and spatiotemporal analysis. *Electroencephalography and Clinical Neurophysiology*, 73(4), 295–305. http://ovidsp.ovid.com/ovidweb.cgi?T=JS&PAGE=reference&D=med3&NEWS=N&AN=2477216	EEG	32	32	na	na	0	32	0	0	0	experiment only	healthy/typical development	1	0	0	1	0	0	0	0	1	0	multiple-session	0	1
H. Halit, M. de H., & Johnson, M. H. (2003). Cortical specialisation for face processing: face-sensitive event-related potential components in 3- and 12-month-old infants. *NeuroImage*, 19(3), 1180–1193. http://ovidsp.ovid.com/ovidweb.cgi?T=JS&PAGE=reference&D=med5&NEWS=N&AN=12880843	EEG	153	51	102	artifacts, procedure error, noncompliance	0	0	26	0	0	experiment only	healthy/typical development	1	0	0	1	0	0	0	0	0	1	single-session	0	1
T. Arichi, et al. (2012). Development of BOLD signal hemodynamic responses in the human brain. *NeuroImage*, 63(2), 663–673. https://www.proquest.com/scholarly-journals/development-bold-signal-hemodynamic-responses/docview/1122591371/se-2	fMRI	51	35	16	motion, awoke during scan	10	15	0	0	10	experiment only	healthy/preterm	0	1	0	1	0	0	0	1	0	0	single-session	0	1
W. Xie, B. M. M., & Richards, J. E. (2019). Development of brain functional connectivity and its relation to infant sustained attention in the first year of life. *Developmental Science*, 22(1), 18-Jan. https://www.proquest.com/scholarly-journals/development-brain-functional-connectivity/docview/2064769832/se-2	EEG	69	59	10	noncompliance, motion artifacts	0	59	0	0	0	experiment only	healthy/typical development	1	0	0	1	0	0	0	0	1	1	single-session	0	1
Braddick, O., & Atkinson, J. (2007). Development of brain mechanisms for visual global processing and object segmentation. *Progress in Brain Research*, 164, 151–168. http://ovidsp.ovid.com/ovidweb.cgi?T=JS&PAGE=reference&D=med6&NEWS=N&AN=17920430	EEG	70	70	na	na	0	70	0	0	0	experiment only	healthy/typical development	1	0	0	0	1	1	0	1	0	0	single-session	0	1
S. Alcauter, et al. (2014). Development of thalamocortical connectivity during infancy and its cognitive correlations. *The Journal of Neuroscience : The Official Journal of the Society for Neuroscience*, 34(27), 9067–9075. http://ovidsp.ovid.com/ovidweb.cgi?T=JS&PAGE=reference&D=med11&NEWS=N&AN=24990927	fMRI	143	143	na	na	0	0	143	0	0	experiment only	healthy/typical development	0	1	0	0	1	1	0	0	0	1	multiple-session	0	1
M. Missana, M. G., & Grossmann, T. (2014). Developmental and Individual Differences in the Neural Processing of Dynamic Expressions of Pain and Anger. *PLoS One*, 9(4). https://www.proquest.com/scholarly-journals/developmental-individual-differences-neural/docview/1977747819/se-2	EEG	60	40	20	artifacts, noncompliance	0	20	0	0	20	experiment only	healthy/typical development	1	0	0	1	0	0	0	0	0	1	single-session	0	1
G. Taga, H. W., & Homae, F. (2018). Developmental changes in cortical sensory processing during wakefulness and sleep. *NeuroImage*, 178, 519–530. http://ovidsp.ovid.com/ovidweb.cgi?T=JS&PAGE=reference&D=med15&NEWS=N&AN=29860079	fNIRS	91	91	na	na	0	91	0	0	0	experiment only	healthy/typical development	1	1	0	1	0	0	0	0	1	1	single-session	0	1
Nishiyori, R., Bisconti, S., Meehan, S. K., & Ulrich, B. D. (2016). Developmental changes in motor cortex activity as infants develop functional motor skills. *Developmental Psychobiology*, 58(6), 773–783. https://doi.org/10.1002/dev.21418	fNIRS	34	25	9	artifacts, noncompliance	0	12	13	0	0	experiment only	healthy/typical development	1	0	0	0	1	1	0	1	0	0	single-session	0	1
E. A. Sheehan, L. L. N., & Mills, D. L. (2007). Developmental changes in neural activity to familiar words and gestures. *Brain and Language*, 101(3), 246–259. http://ovidsp.ovid.com/ovidweb.cgi?T=JS&PAGE=reference&D=med6&NEWS=N&AN=17250885	EEG	51	34	17	artifacts, noncompliance	0	0	0	17	17	experiment only	healthy/typical development	1	0	0	1	0	0	0	0	0	1	two-session	0	1
S. Yrttiaho L. Forssman, J. K., & Leppänen, J. M. (2014). Developmental Precursors of Social Brain Networks: The Emergence of Attentional and Cortical Sensitivity to Facial Expressions in 5 to 7 Months Old Infants. *PLoS One*, 9(6). https://www.proquest.com/scholarly-journals/developmental-precursors-social-brain-networks/docview/1540755049/se-2	EEG	125	115	10	prematurity, noncompliance, experimenter error, technical difficulties	0	115	0	0	0	experiment only	healthy/typical development	1	0	0	1	0	0	0	0	0	1	single-session	0	1
M. V. Lombardo, et al. (2015). Different functional neural substrates for good and poor language outcome in autism. *Neuron*, 86(2), 567–577. http://ovidsp.ovid.com/ovidweb.cgi?T=JS&PAGE=reference&D=med12&NEWS=N&AN=25864635	fMRI	103	103	na	na	0	0	103	0	0	experiment only	ASD/Developmental Language Delay	0	1	0	1	0	0	0	0	1	0	single-session	0	1
Braukmann, R., et al. (2018). Diminished socially selective neural processing in 5-month-old infants at high familial risk of autism. *European Journal of Neuroscience*, 47(6), 720–728. https://doi.org/10.1111/ejn.13751	fNIRS	35	29	6	experimenter error, poor data quality, insufficent trial completion	0	29	0	0	0	experiment only	healthy/high risk of ASD	1	0	0	1	0	0	0	0	0	1	single-session	0	1
A. Blasi, et al. (2011). Early specialization for voice and emotion processing in the infant brain. *Current Biology : CB*, 21(14), 1220–1224. http://ovidsp.ovid.com/ovidweb.cgi?T=JS&PAGE=reference&D=med8&NEWS=N&AN=21723130	fMRI	21	21	na	na	0	21	0	0	0	experiment only	healthy/typical development	0	1	0	1	0	0	0	0	1	0	single-session	0	1
N. L. Maitre, et al. (2015). Effects of caffeine treatment for apnea of prematurity on cortical speech-sound differentiation in preterm infants. *Journal of Child Neurology*, 30(3), 307–313. http://ovidsp.ovid.com/ovidweb.cgi?T=JS&PAGE=reference&D=med12&NEWS=N&AN=24939976	EEG	57	45	12	na	0	45	0	0	0	experiment only	healthy/typical development	0	1	0	1	0	0	0	0	1	0	single-session	0	1
R. Xiao J. Shida-Tokeshi, D. L. V., & Smith, B. A. (2018). Electroencephalography power and coherence changes with age and motor skill development across the first half year of life. *PLoS One*, 13(1). https://www.proquest.com/scholarly-journals/electroencephalography-power-coherence-changes/docview/1986975061/se-2	EEG	21	21	na	na	0	21	0	0	0	experiment only	healthy/typical development	1	0	0	0	1	1	0	1	0	0	multiple-session	0	1
Dehaene-Lambertz, G., & Pena, M. (2001). Electrophysiological evidence for automatic phonetic processing in neonates. *Neuroreport*, 12(14), 3155–3158. http://ovidsp.ovid.com/ovidweb.cgi?T=JS&PAGE=reference&D=med4&NEWS=N&AN=11568655	EEG	15	16	1	awoke during trial	0	16	0	0	0	experiment only	healthy/typical development	0	1	0	1	0	0	0	0	1	0	single-session	0	1
Berger, A. (2011). Electrophysiological evidence for numerosity processing in infancy. *Developmental Neuropsychology*, 36(6), 668–681. http://ovidsp.ovid.com/ovidweb.cgi?T=JS&PAGE=reference&D=med8&NEWS=N&AN=21761992	EEG	13	13	na	na	0	13	0	0	0	experiment only	healthy/typical development	1	0	0	0	1	1	0	0	1	1	single-session	0	1
S. Lippe M. S. Roy, C. P., & Lassonde, M. (2007). Electrophysiological markers of visuocortical development. *Cerebral Cortex (New York, N.Y. : 1991)*, 17(1), 100–107. http://ovidsp.ovid.com/ovidweb.cgi?T=JS&PAGE=reference&D=med6&NEWS=N&AN=16467566	EEG	120	63	57	noncompliance, artifacts, noise	0	43	0	8	28	experiment only	healthy/typical development	1	0	0	1	0	0	0	0	0	1	single-session	0	1
Q. Zhang Q. Cheng, H. L. X. D., & Tu, W. (2017). Evaluation of auditory perception development in neonates by quantitative electroencephalography and auditory event-related potentials. *PLoS ONE*, 12(9), 11. https://www.proquest.com/scholarly-journals/evaluation-auditory-perception-development/docview/2036879329/se-2	EEG	60	53	7	artifacts	0	53	0	0	0	experiment only	healthy/typical development	1	0	0	1	0	0	0	0	1	0	single-session	0	1
R. Slater, et al. (2010). Evoked potentials generated by noxious stimulation in the human infant brain. *European Journal of Pain (London, England)*, 14(3), 321–326. http://ovidsp.ovid.com/ovidweb.cgi?T=JS&PAGE=reference&D=med8&NEWS=N&AN=19481484	EEG	12	12	na	na	0	12	0	0	0	experiment only	healthy/typical development	1	1	0	0	1	0	1	0	0	0	single-session	0	1
T. A. Stroganova, E. V. O., & Posikera, I. N. (1998). Externally and internally controlled attention in infants: an EEG study. *International Journal of Psychophysiology : Official Journal of the International Organization of Psychophysiology*, 30(3), 339–351. http://ovidsp.ovid.com/ovidweb.cgi?T=JS&PAGE=reference&D=med4&NEWS=N&AN=9834890	EEG	46	15	31	did not meet all trial criteria	0	15	0	0	0	experiment only	healthy/typical development	1	0	0	1	1	0	0	0	0	1	single-session	0	1
S. Conte, et al. (2020). Face-sensitive brain responses in the first year of life. *NeuroImage*, 211, 116602. http://ovidsp.ovid.com/ovidweb.cgi?T=JS&PAGE=reference&D=med18&NEWS=N&AN=32044434	EEG	132	132	na	na	0	132	0	0	0	experiment only	healthy/typical development	1	0	0	1	0	0	0	0	0	1	single-session	0	1
He, C., & Trainor, L. J. (2009). Finding the pitch of the missing fundamental in infants. *The Journal of Neuroscience : The Official Journal of the Society for Neuroscience*, 29(24), 7718–8822. http://ovidsp.ovid.com/ovidweb.cgi?T=JS&PAGE=reference&D=med7&NEWS=N&AN=19535583	EEG	103	69	34	noncompliance, fell asleep	0	69	0	0	10	experiment only	healthy/typical development	1	0	0	1	0	0	0	0	1	0	single-session	0	1
Y. Frank D. Kurtzberg, J. A. K., & H. G. Vaughan, Jr. (1992). Flash and pattern-reversal visual evoked potential abnormalities in infants and children with cerebral blindness. *Developmental Medicine and Child Neurology*, 34(4), 305–315. http://ovidsp.ovid.com/ovidweb.cgi?T=JS&PAGE=reference&D=med3&NEWS=N&AN=1572516	EEG	60	na	na	na	na	na	na	na	na	recommended from physician	lack of spontaneous visual behavior	1	0	0	1	0	0	0	0	0	1	single-session	1	0
H. Weibley, et al. (2021). fNIRS monitoring of infant prefrontal cortex during crawling and an executive functioning task. *Frontiers in Behavioral Neuroscience*, 15, 7. https://www.proquest.com/scholarly-journals/fnirs-monitoring-infant-prefrontal-cortex-during/docview/2575509528/se-2?accountid=14521	fNIRS	20	12	8	noncompliance, experimenter error	0	0	12	0	0	experiment only	healthy/typical development	1	0	0	1	1	1	0	1	1	1	two-session	0	1
L. A. Edwards J. B. Wagner, C. E. S., & Hyde, D. C. (2016). Functional brain organization for number processing in pre-verbal infants. *Developmental Science*, 19(5), 757–769. http://ovidsp.ovid.com/ovidweb.cgi?T=JS&PAGE=reference&D=med13&NEWS=N&AN=26395560	fNIRS	30	13	17	poor signal from hair, equipment failure, artifacts	0	13	0	0	0	experiment only	healthy/typical development	1	0	0	1	0	0	0	0	0	1	single-session	0	1
G. Musacchia, et al. (2017). Active auditory experience in infancy promotes brain plasticity in Theta and Gamma oscillations. *Developmental Cognitive Neuroscience*, 26, 19-Sep. http://ovidsp.ovid.com/ovidweb.cgi?T=JS&PAGE=reference&D=med14&NEWS=N&AN=28436834	EEG	49	49	na	na	0	49	0	0	0	experiment only	healthy/typical development	1	0	0	1	1	0	0	0	1	0	multiple-session (6)	0	1
L. Scheef, et al. (2017). Functional Laterality of Task-Evoked Activation in Sensorimotor Cortex of Preterm Infants: An Optimized 3 T fMRI Study Employing a Customized Neonatal Head Coil. *PloS One*, 12(1), e0169392. http://ovidsp.ovid.com/ovidweb.cgi?T=JS&PAGE=reference&D=med14&NEWS=N&AN=28076368	fMRI	13	8	5	hardware failure, motion artifacts	0	8	0	0	0	experiment only	healthy/typical development	0	1	0	0	1	0	1	0	0	0	single-session	0	1
W. Li, et al. (2013). Functional magnetic resonance imaging of the visual cortex performed in children under sedation to assist in presurgical planning. *Journal of Neurosurgery. Pediatrics*, 11(5), 543–546. http://ovidsp.ovid.com/ovidweb.cgi?T=JS&PAGE=reference&D=med10&NEWS=N&AN=23473057	fMRI	11	na	na							Pre surgical	atypical	0	0	1	1	0	0	0	0	0	1	single-session	1	0
M. Mahmoudzadeh F. Wallois, G. K. S. G., & Dehaene-Lambertz, G. (2017). Functional Maps at the Onset of Auditory Inputs in Very Early Preterm Human Neonates. *Cerebral Cortex (New York, N.Y. : 1991)*, 27(4), 2500–2512. http://ovidsp.ovid.com/ovidweb.cgi?T=JS&PAGE=reference&D=med14&NEWS=N&AN=27102655	EEG	19	19	na	na	19	0	0	0	0	experiment only	preterm but otherwise healthy	0	1	0	1	0	0	0	0	1	0	single-session	0	1
G. Dehaene-Lambertz, S. D., & Hertz-Pannier, L. (2002). Functional neuroimaging of speech perception in infants. *Science (New York, N.Y.)*, 298(5600), 2013–2015. http://ovidsp.ovid.com/ovidweb.cgi?T=JS&PAGE=reference&D=med4&NEWS=N&AN=12471265	fMRI	20	20	na	na	0	20	0	0	0	experiment only	healthy/typical development	0	1	0	1	0	0	0	0	1	0	single-session	0	1
D. C. Hyde C. E. Simon, F. T., & Nikolaeva, J. I. (2018). Functional Organization of the Temporal-Parietal Junction for Theory of Mind in Preverbal Infants: A Near-Infrared Spectroscopy Study. *The Journal of Neuroscience : The Official Journal of the Society for Neuroscience*, 38(18), 4264–4274. http://ovidsp.ovid.com/ovidweb.cgi?T=JS&PAGE=reference&D=med15&NEWS=N&AN=29593053	fNIRS	25	20	5	noncompliance, no usable data	0	20	0	0	0	experiment only	healthy/typical development	1	0	0	1	0	0	0	0	0	1	single-session	0	1
D. Perani, et al. (2010). Functional specializations for music processing in the human newborn brain. *Proceedings of the National Academy of Sciences of the United States of America*, 107(10), 4758–4763. http://ovidsp.ovid.com/ovidweb.cgi?T=JS&PAGE=reference&D=med8&NEWS=N&AN=20176953	fMRI	21	18	3	movement artifacts	0	18	0	0	0	experiment only	healthy/typical development	0	1	0	1	0	0	0	0	1	0	single-session	0	1
P. K. Kuhl S. Coffey-Corina, D. P. J. M. A. E., & Dawson, G. (2013). Brain Responses to Words in 2-Year-Olds with Autism Predict Developmental Outcomes at Age 6. *PLoS One*, 8(5). https://www.proquest.com/scholarly-journals/brain-responses-words-2-year-olds-with-autism/docview/1357024534/se-2	EEG	50	24	26	noncompliance, artifacts, lack of english language at home	na	na	na	na	na	experiment only	social symptoms of ASD	1	0	0	1	0	0	0	0	1	1	single-session	0	1
E. Partanen T. Kujala, M. T., & Huotilainen, M. (2013). Prenatal Music Exposure Induces Long-Term Neural Effects. *PLoS One*, 8(10). https://www.proquest.com/scholarly-journals/prenatal-music-exposure-induces-long-term-neural/docview/1447630435/se-2	EEG	24	21	3	hardware issues, motion artifacts	0	21	0	0	0	experimental	healthy, typical development	1	0	0	1	0	0	0	0	1	0	two-session	0	1
I. C. Lisboa H. Miguel, A. S. S. M. J. A. S., & Pereira, A. F. (2020). Right STS responses to biological motion in infancy - An fNIRS study using point-light walkers. *Neuropsychologia*, 149, 107668. http://ovidsp.ovid.com/ovidweb.cgi?T=JS&PAGE=reference&D=med18&NEWS=N&AN=33137357	fNIRS	30	23	7	not looking enough	0	23	0	0	0	experimental	healthy, typical development	1	0	0	0	1	0	0	0	0	1	single session	0	1
Khrizman, T. P., & Zaitseva, L. M. (1981). Role of neocortical association structures in the spatiotemporal organization of brain potentials in children during perception of sensory stimuli of different modalities. *Neuroscience and Behavioral Physiology*, 11(2), 188–194. http://ovidsp.ovid.com/ovidweb.cgi?T=JS&PAGE=reference&D=med2&NEWS=N&AN=7279206	EEG	25	25	na	na	0	0	0	25	0	experimental	healthy, typical development	1	0	0	1	0	0	0	0	1	1	single session	0	1
D. M. Werchan A. G. E. Collins, M. J. F., & Amso, D. (2016). Role of Prefrontal Cortex in Learning and Generalizing Hierarchical Rules in 8-Month-Old Infants. *The Journal of Neuroscience : The Official Journal of the Society for Neuroscience*, 36(40), 10314–10322. http://ovidsp.ovid.com/ovidweb.cgi?T=JS&PAGE=reference&D=med13&NEWS=N&AN=27707968	fNIRS	30	27	3	noncompliance, hair occlusion	0	27	0	0	0	experimental	healthy, typical development	1	0	0	1	0	0	0	0	1	1	single session	0	1
S. G. Erberich A. Panigrahy, P. F. I. S. M. D. N., & Gilles, F. (2006). Somatosensory lateralization in the newborn brain. *NeuroImage*, 29(1), 155–161. http://ovidsp.ovid.com/ovidweb.cgi?T=JS&PAGE=reference&D=med6&NEWS=N&AN=16112875	fMRI	48	30	18	extra-corporeal membrane oxygenation (ECMO), mild non-specific ventriculomegaly	24	6	0	0	0	experimental	healthy, typical development, preterm	0	0	1	1	0	0	1	0	0	0	single session	0	1
Mento, G., & Valenza, E. (2016). Spatiotemporal neurodynamics of automatic temporal expectancy in 9-month old infants. *Scientific Reports*, 6, 36525. http://ovidsp.ovid.com/ovidweb.cgi?T=JS&PAGE=reference&D=med13&NEWS=N&AN=27811953	EEG	35	29	6	noncompliance, motion artifacts	0	15	0	0	14	experimental	healthy, typical development	1	0	0	1	0	0	0	0	1	1	single session	0	1
N. F. Ramírez, et al. (2017). Speech discrimination in 11-month-old bilingual and monolingual infants: A magnetoencephalography study. *Developmental Science*, 20(1). https://www.proquest.com/scholarly-journals/speech-discrimination-11-month-old-bilingual/docview/1779462498/se-2	MEG	33	21	12	hardware issues, noncompliance	0	0	21	0	0	experimental	healthy, typical development	1	0	0	1	0	0	0	0	1	0	single session	0	1
M. Verriotis L. Jones, K. W. M. L.-D. I. P. H. P. J. M. L. F., & Fitzgerald, M. (2018). The distribution of pain activity across the human neonatal brain is sex dependent. *NeuroImage*, 178, 69–77. https://www.proquest.com/scholarly-journals/distribution-pain-activity-across-human-neonatal/docview/2085979249/se-2	EEG	81	81	0	na	0	81	0	0	0	experimental	healthy, typical development	1	0	0	0	1	0	1	0	0	0	single session	0	1
T. Wilcox A. Hirshkowitz, L. H., & Boas, D. A. (2014). The effect of color priming on infant brain and behavior. *NeuroImage*, 85, 302–313. https://www.proquest.com/scholarly-journals/effect-color-priming-on-infant-brain-behavior/docview/1562145727/se-2	fNIRS	53	39	14	hair occlusion, noncompliance	0	39	0	0	0	experimental	healthy, typical development	1	0	0	1	0	0	0	0	0	1	single session	0	1
A. A. Benasich N. Choudhury, J. T. F. T. R.-B. C. C., & Gou, Z. (2006). The infant as a prelinguistic model for language learning impairments: predicting from event-related potentials to behavior. *Neuropsychologia*, 44(3), 396–411. http://ovidsp.ovid.com/ovidweb.cgi?T=JS&PAGE=reference&D=med6&NEWS=N&AN=16054661	EEG	43	43	na	na	0	0	43	0	0	experimental	healthy, typical development, family history of learning disorders	1	0	0	1	0	0	0	0	1	0	4-sessions	0	1

S. Rigato, T. F., & Johnson, M. H. (2010). The shared signal hypothesis and neural responses to expressions and gaze in infants and adults. *Social Cognitive and Affective Neuroscience*, 5(1), 88–97. http://ovidsp.ovid.com/ovidweb.cgi?T=JS&PAGE=reference&D=med8&NEWS=N&AN=19858107	EEG	40	28	12	hardware problems, noncompliance	0	28	0	0	0	experimental	healthy, typical development	1	0	0	1	0	0	0	0	0	1	single session	0	1
L. J. Powell, B. D., & Saxe, R. (2018). Using individual functional channels of interest to study cortical development with fNIRS. *Developmental Science*, 21(4), 13-Jan. https://www.proquest.com/scholarly-journals/using-individual-functional-channels-interest/docview/2348514018/se-2?accountid=14521	fNIRS	17	16	1	failed to complete enough trials	0	16	0	0	0	experimental	healthy, typical development	1	0	0	1	0	0	0	0	0	1	single session	0	1
T. Gliga, A. V., & Csibra, G. (2010). Verbal labels modulate perceptual object processing in 1-year-old children. *Journal of Cognitive Neuroscience*, 22(12), 2781–2789. http://ovidsp.ovid.com/ovidweb.cgi?T=JS&PAGE=reference&D=med8&NEWS=N&AN=20044900	EEG	31	12	19	noncompliance, artifacts	0	0	12	0	0	experimental	healthy, typical development	1	0	0	1	0	0	0	0	0	1	single session	0	1
E. Nakato, et al. (2009). When do infants differentiate profile face from frontal face? A near-infrared spectroscopic study. *Human Brain Mapping*, 30(2), 462–472. http://ovidsp.ovid.com/ovidweb.cgi?T=JS&PAGE=reference&D=med7&NEWS=N&AN=18095284	fNIRS	35	20	15	noncompliance, failure to complete enough trials, artifacts	0	20	0	0	0	experimental	healthy, typical development	1	0	0	1	0	0	0	0	0	1	single session	0	1
G. Musacchia, et al. (2017). Active auditory experience in infancy promotes brain plasticity in Theta and Gamma oscillations. *Developmental Cognitive Neuroscience,* 26, 19-Sep. http://ovidsp.ovid.com/ovidweb.cgi?T=JS&PAGE=reference&D=med14&NEWS=N&AN=28436834	EEG	49	49	na	na	0	49	0	0	0	experimental	healthy, typical development	1	0	0	1	0	0	0	0	1	0	six-sessions	0	1
R. Pickler S. Sealschott, M. M. S. M. J. T. A. P. S. W. L., & Gao, W. (2017). Using Functional Connectivity Magnetic Resonance Imaging to Measure Brain Connectivity in Preterm Infants. *Nursing Research*, 66(6), 490–495. http://ovidsp.ovid.com/ovidweb.cgi?T=JS&PAGE=reference&D=med14&NEWS=N&AN=29095379	fMRI	7	7	na	na	7	0	0	0	0	experimental	preterm infants	0	1	0	0	1	0	1	0	0	0	single session	0	1
H. Watanabe, F. H., & Taga, G. (2010). General to specific development of functional activation in the cerebral cortexes of 2- to 3-month-old infants. *NeuroImage*, 50(4), 1536–1544. https://www.proquest.com/scholarly-journals/general-specific-development-functional/docview/622136554/se-2	EEG fNIRS	204	40	164	sleepiness, hair, motion artifcacts, probe denial	0	40	0	0	0	experimental	healthy, typical development	1	0	0	1	0	0	0	0	1	1	single session	0	1
Kida, T., & Shinohara, K. (2013). Gentle touch activates the prefrontal cortex in infancy: an NIRS study. *Neuroscience Letters*, 541, 63–66. http://ovidsp.ovid.com/ovidweb.cgi?T=JS&PAGE=reference&D=med10&NEWS=N&AN=23416324	fNIRS	32	32	0	na	0	32	0	0	0	experimental	healthy, typical development	1	0	0	1	0	0	1	0	0	0	single session	0	1
C. M. Kelsey, et al. (2021). Gut microbiota composition is associated with newborn functional brain connectivity and behavioral temperament. *Brain, Behavior, and Immunity*, 91, 472–486. https://www.proquest.com/scholarly-journals/gut-microbiota-composition-is-associated-with/docview/2481864390/se-2?accountid=14521	fNIRS	86	63	23	not enough data, crying, high SNR, bad capping, samples did not meet criteria	0	63	0	0	0	experimental	healthy, typical development	1	0	0	1	0	0	0	0	0	1	single session	0	1
Shekhar, S., et al. (2019). Hemodynamic responses to emotional speech in two-month-old infants imaged using diffuse optical tomography. *Scientific Reports*, 9(1), 4745. https://doi.org/10.1038/s41598-019-39993-7	fNIRS	46	21	25	artifacts	0	21	0	0	0	experimental	healthy, typical development	1	0	0	1	0	0	0	0	1	0	single session	0	1
Farah, R., & Horowitz-Kraus, T. (2019). Increased Functional Connectivity Within and Between Cognitive-Control Networks from Early Infancy to Nine Years During Story Listening. *Brain Connectivity*, 9(3), 285–295. http://ovidsp.ovid.com/ovidweb.cgi?T=JS&PAGE=reference&D=med16&NEWS=N&AN=30777454	fMRI	74	74	0	na	0	0	0	3	71	experimental	healthy, typical development	1	0	0	1	0	0	0	0	1	0	single session	0	1
G. Musacchia S. Ortiz-Mantilla, T. R.-B. C. P. R., & Benasich, A. A. (2015). Infant Auditory Processing and Event-related Brain Oscillations. *Journal of Visualized Experiments : JoVE*, 101, e52420. http://ovidsp.ovid.com/ovidweb.cgi?T=JS&PAGE=reference&D=med12&NEWS=N&AN=26167670	EEG	23	23	0	na	0	23	0	0	0	experimental	healthy, typical development	1	0	0	1	0	0	0	0	1	0	single session	0	1
A. N. Meltzoff R. R. Ramirez, J. N. S. E. L. S. T., & Marshall, P. J. (2018). Infant brain responses to felt and observed touch of hands and feet: an MEG study. *Developmental Science*, 21(5), e12651. http://ovidsp.ovid.com/ovidweb.cgi?T=JS&PAGE=reference&D=med15&NEWS=N&AN=29333688	MEG	71	43	28	technical issues, insufficeint trials	0	43	0	0	0	experimental	healthy, typical development	1	0	0	1	0	0	1	0	0	1	single session	0	1
A. N. Bosseler T. Teinonen, M. T., & Huotilainen, M. (2016). Infant Directed Speech Enhances Statistical Learning in Newborn Infants: An ERP Study. *PLoS One*, 11(9). https://www.proquest.com/scholarly-journals/infant-directed-speech-enhances-statistical/docview/1818680880/se-2	EEG	25	23	2	experimenter error, excessive movement	0	23	0	0	0	experimental	healthy, typical development	1	0	0	1	0	0	0	0	1	0	single session	0	1
A. Diaz, et al. (2019). Infant frontal EEG asymmetry moderates the association between maternal behavior and toddler negative affectivity. *Infant Behavior & Development*, 55, 88–99. https://www.proquest.com/scholarly-journals/infant-frontal-eeg-asymmetry-moderates/docview/2235938561/se-2	EEG	410/320	410/320	0	na	0	410	0	320	0	experimental	healthy, typical development	1	0	0	0	1	0	1	0	1	1	two sessions	0	1
Shimada, S., & Hiraki, K. (2006). Infant's brain responses to live and televised action. *NeuroImage*, 32(2), 930–939. http://ovidsp.ovid.com/ovidweb.cgi?T=JS&PAGE=reference&D=med6&NEWS=N&AN=16679032	fNIRS	30	25	5	crying, slippage of probes	0	25	0	0	12	experimental	healthy, typical development	1	0	0	1	0	0	0	0	0	1	single session	0	1
I. C. Lisboa S. Queiros, H. M. A. S. J. A. S., & Pereira, A. F. (2020). Infants' cortical processing of biological motion configuration - A fNIRS study. *Infant Behavior & Development*, 60, 101450. http://ovidsp.ovid.com/ovidweb.cgi?T=JS&PAGE=reference&D=med18&NEWS=N&AN=32417706	fNIRS	24	17	7	equipment failure, inclusion criteria	0	17	0	0	0	experimental	healthy, typical development	1	0	0	1	0	0	0	0	0	1	single session	0	1
van der Weel, S. B. A., & van der Meer, A. L. H. (2019). Infants' brain responses to looming danger: Degeneracy of neural connectivity patterns. *Ecological Psychology*, 31(3), 182–197. https://www.proquest.com/scholarly-journals/infants-brain-responses-looming-danger-degeneracy/docview/2300264459/se-2?accountid=14521	EEG	25	25	0	na	0	25	0	0	0	experimental	healthy, typical development	1	0	0	1	0	0	0	0	0	1	two sessions	0	1
Y. Hakuno M. Hata, N. N. E. -i. H., & Minagawa, Y. (2020). Interactive live fNIRS reveals engagement of the temporoparietal junction in response to social contingency in infants. *NeuroImage*, 218, 13. https://www.proquest.com/scholarly-journals/interactive-live-fnirs-reveals-engagement/docview/2436430584/se-2?accountid=14521	fNIRS	84	39	45	equipment intolerance, fussiness, experimental error	0	39	0	0	0	experimental	healthy, typical development	1	0	0	0	1	0	0	0	1	1	single session	0	1
G. Dehaene-Lambertz, et al. (2010). Language or music, mother or Mozart? Structural and environmental influences on infants' language networks. *Brain and Language*, 114(2), 53–65. https://www.proquest.com/scholarly-journals/language-music-mother-mozart-structural/docview/756305064/se-2?accountid=14521	fMRI	24	7	17	fusiness, no significant activation	0	7	0	0	0	experimental	healthy, typical development	1	0	0	1	0	0	0	0	1	0	single session	0	1
Richards, J. E. (2000). Localizing the development of covert attention in infants with scalp event-related potentials. *Developmental Psychology*, 36(1), 91–108. http://ovidsp.ovid.com/ovidweb.cgi?T=JS&PAGE=reference&D=med4&NEWS=N&AN=10645747	EEG	40	35	5	fusiness/sleepiness, did not complete	0	22	0	13	0	experimental	healthy, typical development	1	0	0	1	0	0	0	0	0	1	single session	0	1
V. L. Shafer, Y. H. Y., & Wagner, M. (2015). Maturation of cortical auditory evoked potentials (CAEPs) to speech recorded from frontocentral and temporal sites: three months to eight years of age. *International Journal of Psychophysiology* : Official Journal of the International Organization of Psychophysiology, 95(2), 77–93. http://ovidsp.ovid.com/ovidweb.cgi?T=JS&PAGE=reference&D=med12&NEWS=N&AN=25219893	EEG	100	90	10	different paradigm, refused, language impairment	0	90	0	0	41	experimental	healthy, typical development	1	0	0	1	0	0	0	0	1	0	multiple sessions	0	1
K. Cuevas M. M. Swingler, M. A. B. S. M., & Calkins, S. D. (2012). Measures of frontal functioning and the emergence of inhibitory control processes at 10 months of age. *Developmental Cognitive Neuroscience,* 2(2), 235–243. https://www.proquest.com/scholarly-journals/measures-frontal-functioning-emergence-inhibitory/docview/1013495165/se-2	EEG	365	365	0	na	0	365	0	0	0	experimental	healthy, typical development	1	0	0	0	1	0	0	0	0	1	single session	0	1
D. Zhang Y. Chen, X. H., & Wu, Y. J. (2019). Near-infrared spectroscopy reveals neural perception of vocal emotions in human neonates. *Human Brain Mapping*, 40(8), 2434–2448. http://ovidsp.ovid.com/ovidweb.cgi?T=JS&PAGE=reference&D=med16&NEWS=N&AN=30697881	fNIRS	60	60	9	crying	0	60	0	0	0	experimental	healthy, typical development	1	1	0	1	0	0	0	0	1	0	single session	0	1
D. C. Hyde D. A. Boas, C. B., & Carey, S. (2010). Near-infrared spectroscopy shows right parietal specialization for number in pre-verbal infants. *NeuroImage*, 53(2), 647–652. https://www.proquest.com/scholarly-journals/near-infrared-spectroscopy-shows-right-parietal/docview/755395409/se-2	fNIRS	74	36	38	fussiness, improper fit, motion, technical difficulties	0	36	0	0	0	experimental	healthy, typical development	1	0	0	1	0	0	0	0	0	1	single session	0	1
van der Kant S. Biro, C. L., & Huijbregts, S. (2018). Negative affect is related to reduced differential neural responses to social and non-social stimuli in 5-to-8-month-old infants: A functional near-infrared spectroscopy-study. *Developmental Cognitive Neuroscience*, 30, 23–30. http://ovidsp.ovid.com/ovidweb.cgi?T=JS&PAGE=reference&D=med15&NEWS=N&AN=29248823	fNIRS	69	37	32	failed stimulus, unusable data	0	37	0	0	0	experimental	healthy, typical development	1	0	0	1	0	0	0	0	0	1	single session	0	1
W. Xie S. A. McCormick, A. W. L. C. B., & Nelson, C. A. (2019). Neural correlates of facial emotion processing in infancy. *Developmental Science*, 22(3), e12758. http://ovidsp.ovid.com/ovidweb.cgi?T=JS&PAGE=reference&D=med16&NEWS=N&AN=30276933	EEG	337	150	187	fussiness, excessive movement, artifacts, insufficient data	0	150	0	0	0	experimental	healthy, typical development	1	0	0	1	0	0	0	0	0	1	single session	0	1
A. Cristia, et al. (2014). Neural correlates of infant accent discrimination: an fNIRS study. *Developmental Science*, 17(4), 628–635. http://ovidsp.ovid.com/ovidweb.cgi?T=JS&PAGE=reference&D=med11&NEWS=N&AN=24628942	fNIRS	60	48	12	did not fit criteria, insufficient data	0	48	0	0	0	experimental	healthy, typical development	1	0	0	1	0	0	0	0	1	1	single session	0	1
D. Perani, et al. (2011). Neural language networks at birth. *Proceedings of the National Academy of Sciences of the United States of America*, 108(38), 16056–16061. http://ovidsp.ovid.com/ovidweb.cgi?T=JS&PAGE=reference&D=med8&NEWS=N&AN=21896765	fMRI	15	15	0	na	0	15	0	0	0	experimental	healthy, typical development	0	1	0	1	0	0	0	0	0	1	single session	0	1
A. N. Meltzoff, J. N. S., & Marshall, P. J. (2019). Neural representations of the body in 60-day-old human infants. *Developmental Science*, 22(1), e12698. http://ovidsp.ovid.com/ovidweb.cgi?T=JS&PAGE=reference&D=med16&NEWS=N&AN=29938877	EEG	41	25	16	fussiness, artifacts	0	25	0	0	0	experimental	healthy, typical development	1	0	0	1	0	0	1	0	0	0	single session	0	1
D. C. Hyde B. L. Jones, R. F., & Porter, C. L. (2011). Neural signatures of face-voice synchrony in 5-month-old human infants. *Developmental Psychobiology*, 53(4), 359–370. http://ovidsp.ovid.com/ovidweb.cgi?T=JS&PAGE=reference&D=med8&NEWS=N&AN=21271561	EEG	75	37	38	equipment placement, artifacts	0	37	0	0	0	experimental	healthy, typical development	1	0	0	1	0	0	0	0	1	1	single session	0	1
Friedrich, M., & Friederici, A. D. (2008). Neurophysiological correlates of online word learning in 14-month-old infants. *Neuroreport*, 19(18), 1757–1761. http://ovidsp.ovid.com/ovidweb.cgi?T=JS&PAGE=reference&D=med7&NEWS=N&AN=18955904	EEG	38	31	7	artifacts, low attention, failure to complete experiment	0	0	31	0	0	experimental	healthy, typical development	1	0	0	1	0	0	0	0	1	1	single session	0	1
S. Benavides-Varela J.-R. Hochmann, F. M. M. N., & Mehler, J. (2012). Newborn's brain activity signals the origin of word memories. *PNAS Proceedings of the National Academy of Sciences of the United States of America*, 109(44), 17908–17913. https://www.proquest.com/scholarly-journals/newborn-s-brain-activity-signals-origin-word/docview/1230618975/se-2	fNIRS	51	44	7	artifacts, crying, experimental error	0	44	0	0	0	experimental	healthy, typical development	1	0	0	1	0	0	0	0	1	0	single session	0	1
E. Quadrelli E. Roberti, C. T., & Craighero, L. (2019). Observation of the point-light animation of a grasping hand activates sensorimotor cortex in nine-month-old infants. *Cortex; a Journal Devoted to the Study of the Nervous System and Behavior*, 119, 373–385. http://ovidsp.ovid.com/ovidweb.cgi?T=JS&PAGE=reference&D=med16&NEWS=N&AN=31401422	EEG	38	19	19	fussiness, artifacts, technical difficulties	0	19	0	0	0	experimental	healthy, typical development	1	0	0	1	0	0	0	0	0	1	single session	0	1
M. Biondi, D. A. B., & Wilcox, T. (2016). On the other hand: Increased cortical activation to human versus mechanical hands in infants. *NeuroImage*, 141, 143–153. http://ovidsp.ovid.com/ovidweb.cgi?T=JS&PAGE=reference&D=med13&NEWS=N&AN=27417344	fNIRS	107	69	38	procedural problems, insufficient data, fussiness	0	69	0	0	0	experimental	healthy, typical development	1	0	0	1	0	0	0	0	0	1	single session	0	1
D. B. Birtles, et al. (2007). Orientation and motion-specific visual cortex responses in infants born preterm. *Neuroreport*, 18(18), 1975–1979. http://ovidsp.ovid.com/ovidweb.cgi?T=JS&PAGE=reference&D=med6&NEWS=N&AN=18007197	EEG	43	43	0	na	17	26	0	0	0	experimental	healthy, preterm & healthy, typical development	1	0	0	1	0	0	0	0	0	1	single session	0	1
S. Bembich P. Brovedani, G. C. L. T. V. G., & Demarini, S. (2015). Pain activates a defined area of the somatosensory and motor cortex in newborn infants. *Acta Paediatrica (Oslo, Norway : 1992)*, 104(11), e530-3. http://ovidsp.ovid.com/ovidweb.cgi?T=JS&PAGE=reference&D=med12&NEWS=N&AN=26174848	fNIRS	25	25	0	na	0	25	0	0	0	experimental	healthy, typical development	1	0	0	0	1	0	1	0	0	0	single session	0	1
M. Bartocci L. L. Bergqvist, H. L., & Anand, K. J. S. (2006). Pain activates cortical areas in the preterm newborn brain. *Pain*, 122(2), 109–117. http://ovidsp.ovid.com/ovidweb.cgi?T=JS&PAGE=reference&D=med6&NEWS=N&AN=16530965	fNIRS	40	40	0	na	0	40	0	0	0	experimental	healthy, typical development	1	0	0	0	1	0	1	0	0	0	single session	0	1
F. Farzin, C. H., & Norcia, A. M. (2012). Piecing it together: infants' neural responses to face and object structure. *Journal of Vision*, 12(13), 6. http://ovidsp.ovid.com/ovidweb.cgi?T=JS&PAGE=reference&D=med9&NEWS=N&AN=23220577	EEG	25	17	8	insufficient data, artifacts	0	17	0	0	0	experimental	healthy, typical development	1	0	0	1	0	0	0	0	0	1	single session	0	1
Bell, M. A. (2002). Power changes in infant EEG frequency bands during a spatial working memory task. *Psychophysiology*, 39(4), 450–458. https://www.proquest.com/scholarly-journals/power-changes-infant-eeg-frequency-bands-during/docview/619920329/se-2?accountid=14521	EEG	62	62	0	na	0	62	0	0	0	experimental	healthy, typical development	1	0	0	0	1	0	0	0	0	1	single session	0	1
V. LoBue J. A. Coan, C. T., & DeLoache, J. S. (2011). Prefrontal Asymmetry and Parent-Rated Temperament in Infants. *PLoS One*, 6(7). https://www.proquest.com/scholarly-journals/prefrontal-asymmetry-parent-rated-temperament/docview/1306219596/se-2	EEG	23	23	0	na	0	23	0	0	0	experimental	healthy, typical development	1	0	0	1	0	0	0	0	1	1	single session	0	1
N. Rotem-Kohavi, N. V.-B., & Oberlander, T. F. (2021). Prenatal exposure to maternal depression is related to the functional connectivity organization underlying emotion perception in 8-10-month-old infants—Preliminary findings. *Infant Behavior & Development*, 63, 7. https://www.proquest.com/scholarly-journals/prenatal-exposure-maternal-depression-is-related/docview/2544512265/se-2?accountid=14521	EEG	35	30	5	artifacts	0	30	0	0	0	experimental	healthy, typical development	1	0	0	1	0	0	0	0	0	1	single session	0	1
de Haan, M., & Nelson, C. A. (1997). Recognition of the mother's face by six-month-old infants: a neurobehavioral study. *Child Development*, 68(2), 187–210. http://ovidsp.ovid.com/ovidweb.cgi?T=JS&PAGE=reference&D=med4&NEWS=N&AN=9179998	EEG	276	120	156	artifacts, fussiness, excessive movement, equipment failure	0	120	0	0	0	experimental	healthy, typical development	1	0	0	1	0	0	0	0	0	1	single session	0	1
C. T. Ellis, et al. (2021). Retinotopic organization of visual cortex in human infants. *Neuron*, 109(16), 2616-2626.e6. http://ovidsp.ovid.com/ovidweb.cgi?T=JS&PAGE=reference&D=medl&NEWS=N&AN=34228960	fMRI	17	12	5	head motion, insufficient data	0	0	12	0	0	experimentla	healthy, typical development	1	0	0	1	0	0	0	0	0	1	single session	0	1
Khrizman, T. P., & Zaitseva, L. M. (1981). Role of neocortical association structures in the spatiotemporal organization of brain potentials in children during perception of sensory stimuli of different modalities. *Neuroscience and Behavioral Physiology*, 11(2), 188–194. http://ovidsp.ovid.com/ovidweb.cgi?T=JS&PAGE=reference&D=med2&NEWS=N&AN=7279206	EEG	25	25	0	na	0	0	0	25	0	experimental	healthy, typical development	1	0	0	1	0	0	0	0	1	1	single session	0	1
S. Urakawa K. Takamoto, A. I. T. O., & Nishijo, H. (2015). Selective medial prefrontal cortex responses during live mutual gaze interactions in human infants: An fNIRS study. *Brain Topography*, 28(5), 691–701. https://www.proquest.com/scholarly-journals/selective-medial-prefrontal-cortex-responses/docview/1627954850/se-2?accountid=14521	fNIRS	18	11	7	crying, motion artifacts	0	11	0	0	0	experimental	healthy, typical development	1	0	0	0	1	0	0	0	0	1	single session	0	1
J. A. Hamalainen, S. O.-M., & Benasich, A. A. (2011). Source localization of event-related potentials to pitch change mapped onto age-appropriate MRIs at 6 months of age. *NeuroImage*, 54(3), 1910–1918. http://ovidsp.ovid.com/ovidweb.cgi?T=JS&PAGE=reference&D=med8&NEWS=N&AN=20951812	EEG	55	39	16	noise, neurological problems	0	39	0	0	0	experimental	healthy, typical development	1	0	0	1	0	0	0	0	1	0	single session	0	1
K. E. Travis, et al. (2011). Spatiotemporal neural dynamics of word understanding in 12- to 18-month-old-infants. *Cerebral Cortex (New York, N.Y. : 1991)*, 21(8), 1832–1839. http://ovidsp.ovid.com/ovidweb.cgi?T=JS&PAGE=reference&D=med8&NEWS=N&AN=21209121	MEG	12	12	0	na	0	0	0	12	0	experimental	healthy, typical development	1	0	0	1	0	0	0	0	1	1	single session	0	1
G. Taga, H. W., & Homae, F. (2011). Spatiotemporal properties of cortical haemodynamic response to auditory stimuli in sleeping infants revealed by multi-channel near-infrared spectroscopy. *Philosophical Transactions. Series A, Mathematical, Physical, and Engineering Sciences*, 369(1955), 4495–4511. http://ovidsp.ovid.com/ovidweb.cgi?T=JS&PAGE=reference&D=med8&NEWS=N&AN=22006903	fNIRS	38	18	20	awoke, motion artifacts	0	18	0	0	0	experimental	healthy, typical development	0	1	0	1	0	0	0	0	1	0	single session	0	1
Dehaene-Lambertz, G., & Dehaene, S. (1994). Speed and cerebral correlates of syllable discrimination in infants. *Nature*, 370(6487), 292–295. http://ovidsp.ovid.com/ovidweb.cgi?T=JS&PAGE=reference&D=med3&NEWS=N&AN=8035876	EEG	23	16	7	insufficient data	0	16	0	0	0	experimental	healthy, typical development	1	0	0	1	0	0	0	0	1	0	single session	0	1

T. Grossmann, E. P., & Friederici, A. D. (2010). The detection of communicative signals directed at the self in infant prefrontal cortex. *Frontiers in Human Neuroscience*, 4, 5. https://www.proquest.com/scholarly-journals/detection-communicative-signals-directed-at-self/docview/845399513/se-2	fNIRS	29	20	9	fusiness, motion artifacts, technical issues	0	20	0	0	0	experimental	healthy, typical development	1	0	0	1	0	0	0	0	1	1	single session	0	1
S. Lloyd-Fox, et al. (2012). The emergence of cerebral specialization for the human voice over the first months of life. *Social Neuroscience*, 7(3), 317–330. https://www.proquest.com/scholarly-journals/emergence-cerebral-specialization-human-voice/docview/1015244183/se-2?accountid=14521	fNIRS	50	33	17	insufficient data, technical issues, hair	0	33	0	0	0	experimental	healthy, typical development	1	0	0	1	0	0	0	0	1	1	single session	0	1
F. Homae H. Watanabe, T. N. K. A., & Taga, G. (2006). The right hemisphere of sleeping infant perceives sentential prosody. *Neuroscience Research*, 54(4), 276–280. http://ovidsp.ovid.com/ovidweb.cgi?T=JS&PAGE=reference&D=med6&NEWS=N&AN=16427714	fNIRS	31	21	10	awoke, motion artifacts, experimental error	0	21	0	0	0	experimental	healthy, typical development	0	1	0	1	0	0	0	0	1	0	single session	0	1
T. Correia, et al. (2012). Three-dimensional optical topography of brain activity in infants watching videos of human movement. *Physics in Medicine and Biology*, 57(5), 1135–1146. http://ovidsp.ovid.com/ovidweb.cgi?T=JS&PAGE=reference&D=med9&NEWS=N&AN=22330053	fNIRS	13	13	0	na	0	13	0	0	0	experimental	healthy, typical development	1	0	0	0	1	0	0	0	0	1	single session	0	1
A. N. Bosseler, et al. (2021). Using magnetoencephalography to examine word recognition, lateralization, and future language skills in 14-month-old infants. *Developmental Cognitive Neuroscience*, 47, 10. https://www.proquest.com/scholarly-journals/using-magnetoencephalography-examine-word/docview/2490709125/se-2?accountid=14521	MEG	32	27	5	diagnosis of speech delay, excessive movement, equipment malfunction	0	27	0	0	0	experimental	healthy, typical development	1	0	0	1	0	0	0	0	1	0	single session	0	1
D. C. Hyde B. L. Jones, C. L. P., & Flom, R. (2010). Visual stimulation enhances auditory processing in 3-month-old infants and adults. *Developmental Psychobiology*, 52(2), 181–189. http://ovidsp.ovid.com/ovidweb.cgi?T=JS&PAGE=reference&D=med8&NEWS=N&AN=20014224	EEG	56	30	26	fussiness, failed to attend	0	16	0	0	14	experimental	healthy, typical development	1	0	0	1	0	0	0	0	1	1	single session	0	1
S. Hoehl, L. W., & Striano, T. (2008). Young Infants' Neural Processing of Objects Is Affected by Eye Gaze Direction and Emotional Expression. *PLoS One*, 3(6). https://www.proquest.com/scholarly-journals/young-infants-neural-processing-objects-is/docview/1312288790/se-2	EEG	73	15	58	fussiness, insufficient data, artifacts	0	15	0	0	0	experimental	healthy, typical development	1	0	0	1	0	0	0	0	0	1	single session	0	1
P. K. Kuhl S. Coffey-Corina, D. P. J. M. A. E., & Dawson, G. (2013). Brain Responses to Words in 2-Year-Olds with Autism Predict Developmental Outcomes at Age 6. *PLoS One*, 8(5). https://www.proquest.com/scholarly-journals/brain-responses-words-2-year-olds-with-autism/docview/1357024534/se-2	EEG	50	24	26	insufficient data, equipment, English not primarly language, declined ERP testing, seizure	0	0	24	0	0	experimental	children with ASD	1	0	0	1	0	0	0	0	1	0	single session	0	1
