# Supplementary material for: Task-based functional neuroimaging in infants: a systematic review
Source: Front Neurosci. 2023 Aug 16;17:1233990. doi: 10.3389/fnins.2023.1233990 (PMC10466897; doi:10.3389/fnins.2023.1233990)
Supplement: Supplementary file 2 [file Table_1.docx]

**APPENDIX 1**

**Search Strategy and Results**

The search used five major databases to identify articles related to fUS in infants: PubMed, Medline OVID, IEEE Xplore, Web of Science, and four databases provided through ProQuest (APA PsycArticles, APA PsycExtra, APA PsycInfo, and . PubMed and Medline @OVID index biomedical and life sciences literature, and both allow robust searching using MeSH terms. Web of Science indexes all sciences, including health sciences, and includes conference proceedings. IEEE Xplore contains material, including conference proceedings, related to electrical engineering, electronics, and their allied fields; IEEE Xplore was selected since the question is interdisciplinary in nature, and related to diagnostic equipment. The database searches were conducted on October 16, 2021, with the exception of IEEE Xplore. IEEE Xplore was searched October 19, 2021, as it was offline at the time of initial search. The search strategies explicitly included all dates available, up to and including October 15, 2021. Since the search terms were very specific to fUS on infants involving the cerebrum or cerebellum, article types were not filtered. The combination of the five databases provides a comprehensive search method, addressing the search from multiple vantage points and disciplines. Specific search phrases and nuances particular to each database are below. Search results were exported from each database, and imported to a dedicated EndNote library. The articles were deduplicated using EndNote's deduplication function, then manually by computationally matching for similarities using titles (stripped of all non-alphanumeric characters), the first 50 characters of the abstracts (stripped of all non-alphanumeric characters), and the article volume+number. There were 854 articles after deduplicating the initial 1118 citations.

**PubMed**

The National Library of Medicine’s PubMed contains millions of citations to biomedical literature. The value of using PubMed, beyond its large number of citations, is the articles are evaluated by librarians who add MeSH terms to the metadata. These MeSH terms increase the findability and accuracy of articles returned by searches. The PubMed search was completed using the Advanced Search option. No additional filters were used. The search returned 234 results.

Search phrase

"functional neuroimaging"[MeSH Terms] AND "Analytical, Diagnostic and Therapeutic Techniques and Equipment Category"[MeSH Major Topic] AND "infant"[MeSH Terms] AND ("Cerebrum"[Mesh Major Topic] OR "Cerebellum"[Mesh Major Topic]) AND ("0001/01/01"[PDAT] : "2021/10/15"[PDAT])

Limit to English Language

**Medline @OVID**

The resource option selected in Ovid was *Ovid MEDLINE(R) and In-Process & Other Non-Indexed Citations 1946 to October 15 2021.* Ovid allows the ability to apply a date range limit after the initial search using certain fields. Since the month and day are not always present as part of the date published, a granular date range (i.e. a range that is more than just the year), must rely on a different field. The Entrez Date field (ez) was chosen for this limit as it is the date the citation was added to PubMed, and most accurately reflects the date of publication. The search returned 587 results.

Search phrase (Advanced Search)

1 (exp diagnostic imaging/ or exp diagnostic techniques, neurological/) and exp Infant/ and (exp Cerebrum/ or exp Cerebellum/) and exp Functional Neuroimaging/

2 limit 1 to (english language and ez=19460101-20211015)

**IEEE Xplore**

While IEEE Xplore allows advanced search using MeSH Terms, a search using the “All Metadata” field was selected to allow the broadest return and inclusion of relevant articles. Using a command search, the ability to apply a year published filter occurs after the initial search, and thus is limited by the initial search results. In this case, while the search was conducted on October 19, 2021, the results were inclusive of the year range, 1994-2019. The search returned 14 results.

Search phrase (Command Search)

(("All Metadata": cerebrum OR "All Metadata": cerebellum) ) AND (infant* OR newborn)

**ProQuest: APA PsycArticles, APA PsycExtra, and APA PsycInfo**

The APA databases use APA Thesaurus of Psychological Index Terms - Summer 2021, a hierarchical vocabulary. This allows the search to "explode" a subject term and obtain results associated with narrower terms. The search returned 140 results.

Search Strategy (Command Line Search with subsequent limiters)

(MAINSUBJECT.EXACT.EXPLODE("Infant Development") AND MAINSUBJECT.EXACT.EXPLODE("Brain") AND (MAINSUBJECT.EXACT.EXPLODE("Medical Diagnosis") OR MAINSUBJECT.EXACT.EXPLODE("Neuroimaging")) AND function*)

Additional limits - Peer reviewed, Date: Before October 15 2021; Language: English

**ProQuest: Materials Science & Engineering Collection‎**

ProQuests Materials Science & Engineering Collection‎ is an interdisciplinary engineering database. Indexing in the collection is supported by a controlled vocabulary. The search strategy combined main subject terms with a general text search term. The search returned 117 results.

Search Strategy (Command Line Search with subsequent limiters)

MAINSUBJECT.EXACT("babies") AND MAINSUBJECT.EXACT("brain research") AND function*

Limit to english language, peer reviewed, publication dates end October 15, 2021

Exclude wire feeds

**Web of Science**

Web of Science provides an interface to search multiple databases. It does not allow granular search of dates, but only year published. The Web of Science search included all databases available with our particular subscription, but results were from the following databases: Web of Science Core Collection, BIOSIS Citation Index, Current Contents Connect, MEDLINE®,

BIOSIS Previews. Since Web of Science does not allow searching by MeSH terms, the search phrase was created using the Topic field tags. The search returned 26 results.

Search phrase (Advanced Search)

TS=("functional neuroimaging" AND (infant* OR newborn)) AND TS=(cerebrum OR cerebellum)
